# Supplementary material for: Parallel reduction in flowering time from de novo mutations enable evolutionary rescue in colonizing lineages
Source: Nat Commun. 2022 Mar 18;13:1461. doi: 10.1038/s41467-022-28800-z (PMC8933414; doi:10.1038/s41467-022-28800-z)
Supplement: Supplementary file 1 — Supplementary Information [file 41467_2022_28800_MOESM1_ESM.pdf]

Parallel reductions in flowering time from *de novo* mutations enable evolutionary rescue in colonizing lineages

Fulgione and Neto *et al.*

Supplementary Method 1. Sample collection

We collected plants over a series of field expeditions between 2012 and 2019 on Santo Antão and Fogo, the two islands where *Arabidopsis thaliana* had been recorded in herbarium records (personal communication, Wolfram Lobin). In addition, we explored possible locations in the two other islands with the most similar landscape (Santiago and São Nicolau) but found no evidence of *A. thaliana* there, consistent with a lack of herbarium records. In total, we present data for 335 accessions from the Cape Verde Islands (Supplementary Data 1), including 189 accessions from 26 stands across four regions in Santo Antão (Cova de Paúl, Lombo de Figueira, Pico da Cruz and Espongeiro), and 146 accessions from 18 stands across three regions in Fogo (Lava, Monte Velha and Inferno).

Supplementary Method 2. Climate data

We downloaded gridded data for climatic and bioclimatic variables at 30 second resolution (~ 1 km^2) in GeoTiff file format for the temporal range of 1970-2000 from WorldClim version 2.1[1], including monthly climate data for average temperature and precipitation (12 data layers, i.e. 1 layer for each month) and 19 bioclimatic variables (1 data layer each) which are temperature and rainfall derived datasets. In addition, we downloaded a raster file for Aridity Index (1 data layer) from CGIAR Consortium for Spatial Information (CGIAR-CSI)[2], which is the ratio of precipitation to potential evapotranspiration, where higher values correspond to more humid conditions and lower to more arid conditions. In addition, we estimated growing season length

using the monthly average temperature and precipitation data obtained from WorldClim. Months for which mean temperature ≥ 4°C and mean precipitation ≥ 2 * mean temperature were summed to produce an estimate of the growing season length[3] using 'Raster Calculator' of ArcGIS. We extracted values for sites where CVI, Moroccan and Eurasian samples had been collected in ArcGIS and compared distributions of climate variables across regions using Mann Whitney Wilcoxon (MWW) tests.

Supplementary Method 3. Sequencing

We sequenced 335 newly collected Cape Verde Islands accessions and Cvi-0 using Illumina Hi-Seq and HiSeq3000 machines. We extracted genomic DNA using DNeasy Plant Mini kits (Qiagen), fragmented using sonication (Covaris S2), and prepared libraries with Illumina TruSeq DNA sample prep kits (Illumina), NEBNext Ultra II FS DNA Library Prep Kit (New England Biolabs) and NEBNext Ultra II DNA Library Prep Kit (New England Biolabs). Libraries were immobilized and processed onto a flow cell with cBot (Illumina) and subsequently sequenced with 2x 100-150 bp paired end reads. We assessed DNA quality and quantity via capillary electrophoresis (TapeStation, Agilent Technologies) and fluorometry (Qubit and Nanodrop, Thermo Fisher Scientific). Due to changes in product availability over time, sample preparation differed slightly between subsets of the sequenced accessions. Sample IDs 12766 to 35519 were prepared with Illumina TruSeq DNA sample prep kits (Illumina, San Diego, CA), samples in projects 4073 and 3968 were prepared with NEBNext Ultra II FS DNA Library Prep Kits (Illumina, New England Biolabs) including four cycles of PCR amplification, and samples from

projects 3619, 3541, 3536, and 2876 were prepared with NEBNext Ultra II DNA Library Prep Kit (Illumina, New England Biolabs) with five cycles of PCR amplification.

Supplementary Method 4. SNP identification and genotyping

For Illumina sequence data mapping and genotype calling we used the *A. thaliana* TAIR10 reference genome and we called variants with three different pipelines. Two pipelines were previously used to call genotypes in the 1135 Eurasian and 64 Moroccan sequences[4]. Here, we used the same parameters, settings, and software versions in order to analyse the Cape Verdean and worldwide sequences in a common framework (https://github.com/HancockLab/CVI). Specifically, in the SHORE pipeline[5] we pre-processed the reference genome with the subprogram ‘preprocess’ and parameters < -C --indexes BWA,SuffixArray > and with bwa v0.7.5a[6], command ‘index’, parameters < -a bwtsw >. We trimmed adapters with adapterremoval v2.1.2[7], parameters < --trimns –trimqualities >. We aligned reads to the reference genome with the bwa program ‘aln’, parameters < -n 0.1 > and ‘sample’, parameter < -a 500 >, and we imported trimmed fastq files using the SHORE subprogram ‘import’ with parameters < --application genomic --importer Fastq --shore-filter --max-Ns 10% --lowcomplexity >. Finally, we called variants with the empirical scoring matrix approach implemented in the SHORE subprogram ‘consensus’ with parameters < -b 0.9 -g 4 -h 6 -i 0.5 -N >. We used this pipeline for all analyses except MSMC, GWAS and BSA. The second, more conservative pipeline is implemented as a custom program in java v.1.8, and it was used to reduce false positive variant calls due to indels for the MSMC analysis, which is sensitive to linked errors[4]. This pipeline

excludes repetitive genomic regions, such as regions where the same base is repeated five or more times, as well as the adjacent ten bases, it excludes the first and last positions of each read, it removes bases with quality < 30 and coverage < 5x, and it eliminates positions with coverage greater than twice the average coverage to remove potential duplications. For variant calling, the pipeline calls the reference allele with a calling ratio of 0.0 to 0.2, and a mismatch to the reference with a calling ratio of 0.8 to 1.0. To avoid strand-specific errors, mismatches are called only if they are supported by at least one read aligned on both the forward and reverse strand. To call short indels, we used a modified version of the GATKv.4.1.3.0[8] best practices workflow for germline short variant discovery and genotyping. We included biallelic variants only and converted heterozygous sites to missing data to mask possible false positives. Further, we converted genomic regions with coverage higher than twice the genomic average to missing data, to eliminate false variant calls due to duplications not represented in the reference genome. In the three pipelines, file conversions between fastq, bam and sam formats relied on picard v. 2.21.1 and samtools v. 1.9[9], vcf merging and subsetting relied on bcftools vl.9[10] and vcftools v. 0.1.14[11]. Average coverage across samples was 19.4x (range from 9.3x to 51.7x; Supplementary Data 1) after alignment to the reference.

For all downstream analyses, we retained variants with coverage greater than 3 and base quality greater than 25 in the SHORE calls, and 2 and 30 in the GATK calls (https://github.com/HancockLab/CVI). To call S-locus haplogroups we followed the procedure used in [4]. We added to the reference genome (TAIR10), which represents haplogroup A, the

sequences of S-locus haplogroups B and C (from Cvi-0[12] and Lz-0[13], respectively). We called variants against this modified reference and assigned to each CVI sample the S-locus haplogroup that had the highest proportion of sites with non-zero coverage, after quality filtering.

Supplementary Method 5. Plant growth and phenotyping

In the flowering time experiment, we scored flowering time, bolting time, time to anthesis, number of days until the stem reached 3 cm, and the number of rosette leaves at bolting, as in Salomé *et al.* [14]. We measured correlation between the four flowering traits scored in the simulated CVI conditions experiment using the function cor() implemented in R (https://github.com/HancockLab/CVI). These phenotypes were strongly correlated, with Spearman's rho of at least 0.96 in Santo Antão and Morocco between flowering time and bolting time. In Fogo, where flowering time is more difficult to score due to differences in petal morphology, the correlation was somewhat lower (rho=0.83), likely due to increased error for the flowering time trait here. Therefore, bolting time results were used as a proxy for flowering time in downstream analyses.

Supplementary Method 6. Linkage disequilibrium

Linkage disequilibrium (LD) was assessed between pairs of SNPs with minor allele frequency greater than 5% were calculated using the command < --ld-window 999 --ld-window-kb 10 --ld-window-r2 0 --r2 --snps-only > in PLINK[15]. The numbers of sites on which LD was

estimated were 55, 645 for Santo Antão, 56, 173 for Fogo and 1, 435, 763 for Morocco. Calculations were made between pairs of SNPs up to a distance of 10 kb. LD decay analyses were conducted by division of marker pairs within the 10-kb region into bins of 1 kb and r^2 values within each bin were averaged. To visualize the result, the r^2 values were sorted and plotted against the physical distance, using loess smoothing.

Supplementary Method 7. Demographic reconstruction

Using MSMC-CCR[16], we inferred split times by computing the mean across combinations of sets of samples and the confidence interval of the mean (±1.96*standard error of the mean). For the split between Santo Antão and Fogo, we used a total of 63 combinations of eight accessions from each island. For the split to Morocco, we used 357 combinations (separately for the High Atlas, South and North Middle Atlas Moroccan populations). For splits within Santo Antão, we used 12 combinations to examine pairwise splits between the Figueira, Cova, Espongeiro, and Pico da Cruz populations. As suggested in [16], we inferred split times when CCR reached 0.5, with an uncertainty interval between $0.25 \leq CCR \leq 0.75$.

For the inference of split parameters in dadi v.2.1.0[17], we used joint site frequency spectra (JSFS) based on intergenic SNPs, which are less likely to evolve under strong selection than coding regions. We estimated parameters between the two Cape Verde islands and between CVI and Morocco using four demographic models: 1) a simple two-population split model with no

migration and constant population size (N_e); 2) the same model with a bottleneck at the split; 3) a split with exponential changes in N_e after the split and no migration; and 4) an isolation with migration model (IM): a split with exponential changes in N_e and asymmetric migration.

For each demographic model and population pair, we replicated the analysis 1000 times with a maximum of 50 iterations. In each replicate run we used starting values for all parameters drawn randomly from predefined ranges. The parameter boundaries were ($10^{-3}*N_{ref}$; $20*N_{ref}$) for effective population sizes (N_e), (0; $20/N_{ref}$) for migration rate, and (0; $10*N_{ref}$) for the split time, where N_{ref} is the size of the ancestral population. Among the 1000 runs per model, we selected the parameter combination that resulted in the highest likelihood. We identified the model with the best support using the Akaike information criterion (AIC), and for each resulting best model, we calculated confidence intervals for parameters using 100 000 bootstrapped data sets and the Godambe Information Matrix implemented in dadi. For the best-supported models, the 5% runs with highest likelihood all converged to the same parameter set.

We inferred colonization time by obtaining an upper bound based on the minimum coalescence time between CVI and Morocco, and a lower bound based on the maximum coalescence time within the CVI clade. First, we ran coalescent simulations of the CVI-Moroccan split in msprime v.0.4.0[18] with split times drawn from a uniform distribution of times between 5-50 kya. To account for the confounding effect of purifying selection, which reduces the rate at which new mutations are introduced in the genome, we scaled mutation rate across

simulated genomic windows as $\mu_{scaled}=\theta_{local}/4*N_e$, where θ_{local} was estimated as θ_π in the Moroccan population within each window and N_e was fixed to the genome-wide average ($N_e=\theta_{genome}/4*\mu$) so that $\mu_{scaled}=\mu*(\theta_{local}/\theta_{genome})$. Then, we inferred coalescence times between simulated and observed CVI and Moroccan genomes across genomic windows based on the density of mutations. We obtained 95% confidence intervals based on the standard error (SE) estimated by non-parametric bootstrap resampling of observed and simulated data. By fitting the simulated cumulative proportion of genomic windows with different inferred ages to observed data, we obtained a conservative estimate of the upper bound of colonization time. We inferred coalescence times within Cape Verde, across genomic windows (0.1 Mbp, non-overlapping), based on the density of mutations and used the 95th percentile as a lower bound for colonization time.

We constructed a time-calibrated chloroplast phylogeny to examine divergence in the chloroplast genome and to compare these to patterns at nuclear loci. First, we aligned the chloroplast sequences with outgroups from other *Arabidopsis* species, *Capsella grandiflora*, *Capsella bursa-pastoris* and *Camelina sativa*[19], excluding the Inverted Repeat region. All indels were removed. Identical sequences were excluded from the alignment and a maximum likelihood (ML) phylogenetic tree was reconstructed with RAxML v.8.1.16 [20] using the GTR+Γ+I model of rate heterogeneity and setting the clade of *Capsella* and *Camelina* as outgroup. Rapid bootstrapping followed by a thorough ML search was applied with 1000 bootstrap replicates.

Divergence time was estimated using BEAST v.1.8.3[21]. Three secondary calibration points were included from the literature[22]: the root height (split between genus *Arabidopsis* and the *Capsella*/*Camelina* clade) was set to 8.1627 million years (my), the split between *Capsella* and *Camelina* was set to 7.3572 my, and the crown age of genus *Arabidopsis* was set to 5.9685 my; a standard deviation of 1.0 was used for all three calibration points. The GTR+Γ+I model of rate heterogeneity with 4 Gamma categories was used as substitution model with an uncorrelated relaxed lognormal clock[23] and tree prior Speciation: Birth-Death Process[24]. Two independent MCMC runs with chain length $1x10^9$ were combined in LogCombiner v.1.8.3[21], discarding the first 10% of each run as burn-in, and the median heights from the remaining 18002 trees were annotated onto the maximum clade credibility tree in TreeAnnotator v.1.8.3[21].

Supplementary Method 8. Additional inference of demography within CVI

We used forward, individual-based simulations in SLiM v.3.3.2[25] to model the demographic history within the archipelago, including the colonization events and consequent bottlenecks. Under this model, the initial propagule founded a population on one island in the archipelago. The population grew following an exponential function that varies across simulations (growth rate varies with final size between 100 and 2500 individuals) until the time of the split between islands (4.0 kya), when the second island was colonized from the first. Both populations grew exponentially until they reached final N_e (10K, inferred from θ_π). On the second island, as inferred with dadi[17], we simulated a 1000 year-long bottleneck with 400 individuals.

In order to determine which island was colonized first, we fit simulations to the observed data using the difference in the proportion of variants that are fixed in one island and segregating in the other (proportion of variants segregating in Santo Antão and fixed in Fogo minus the proportion of variants segregating in Fogo and fixed in Santo Antão) as a summary statistic. The value of this statistic is positive if Santo Antão was colonized first and negative if Fogo was colonized first. In addition, we used three-way (Morocco, Santo Antão, Fogo) JSFS modelling in dadi to compare the relative fit of Santo Antão-first and Fogo-first models and estimated the length of the Fogo bottleneck period[17]. For each demographic model we replicated the analysis 100 times with a maximum of 50 iterations. In each replicated run, we used starting values for all parameters drawn randomly from predefined ranges. The parameter boundaries were (10^{-3}*N_{ref}; 20*N_{ref}) for effective population sizes (N_e), and (0; 10*N_{ref}) for split times, where N_{ref} is the size of the ancestral population. We identified the model with the best support using the Akaike information criterion (AIC).

Supplementary Method 9. Niche modelling

In the first step of niche modelling in MaxEnt[26], we produced a predictive model using collection locations in Morocco and the bioclimatic variables described above and listed in Supplementary Data 2. We considered supplementing the collection locations with information from herbarium collection records from GBIF (https://www.gbif.org), but only ‘fuzzy matches’

existed in the data base and GPS coordinates were thus unreliable. We conducted climatic niche modelling in Morocco using occurrence data based on[27]. Data for the Moroccan region was extracted using the 'extract by mask' function in ArcToolbox. To avoid overfitting, climatic variables were pruned so that no two variables were correlated with Pearson correlation coefficient > 0.75. The model we present uses a set of variables chosen based on ecological and biological relevance, but the predicted suitability of CVI habitat for Moroccan accessions did not change across these different variable selection regimes. We ran a Maxent under the standard default parameters with jackknife resampling to estimate the importance of each variable on the model. Model fit was inferred based on the area under the curve (AUC) for the model output and a cross-validation approach in which the data were split into equal sized subsets. Then, we projected this model onto the CVI landscape to predict the suitable range of Moroccan samples in CVI. We further identified the regions within CVI that were most similar to the Moroccan *A. thaliana* habitat. Since the approach we used for pruning variables is somewhat subjective and the CVI suitability result was extreme, we also tried other approaches for pruning correlated variables but found no change in suitability in CVI across variable selection regimes including random selection of variables with Pearson's $r < 0.75$ and a variance inflation factor approach. This robustness is likely due to the fact that nearly all climate variable values for CVI lie outside those found in the Moroccan presence data.

Supplementary Method 10. Testing for evidence of adaptive evolution

To compute the d_{sel}/d_{neu} ratio, we used custom scripts written in java v.1.8 (https://github.com/HancockLab/CVI) and defined it as the rate ratio of 0-fold non-synonymous to 4-fold synonymous substitutions, scaled by the number of sites at risk for each category as in the following equation.

$$\frac{\text{dsel}}{\text{dneu}} = \frac{\frac{\text{number of 0-fold substitutions}}{\text{number of sites at risk for 0-fold substitutions}}}{\frac{\text{number of 4-fold substitutions}}{\text{number of sites at risk for 4-fold substitutions}}} \quad (1)$$

For this, we first constructed an artificial variant call format (VCF) file with all possible variants at all sites in the genome and annotated them with SnpEff v.3.0.7[28]. Then we used a custom script for the calculation of JSFS (https://github.com/HancockLab/CVI) to compute the number of zero- and four-fold degenerate substitutions, as proxies for selected and neutral sites, respectively. Note that this is a simplified approach to estimating dN/dS, which excludes 2- and 3-fold degenerate sites. We used this approach because estimating the expected changes at these classes of sites is problematic due to asymmetries in substitution rates. We scaled these to the number of sites in the genome at risk for each substitution type and deducted the positions with more than 5% missing data. For continental clades, the spectra were polarized to *A. lyrata* samples. Due to the long divergence time and genomic rearrangements between species, the alignment of *A. lyrata* to *A. thaliana* reduced the number of bases for the analyses to 70676280. For the CVI populations, we defined substitutions as variants derived in comparison to Morocco, fixed in one island and absent from the other. To estimate uncertainty, we bootstrapped frequency spectra 500 times in polyDfe v.2.0[29] and calculated empirical *p*-values based on the bootstrapped data. The large variance in the bootstrapped data stems from the low number of

total variants fixed in the two island populations. If the number of four-fold substitutions was zero (in real or bootstrapped data), we conservatively added one to avoid dividing by zero. For the Moroccan clade, we used *Arabidopsis lyrata* samples as an outgroup. We used the spectra at zero- and four-fold degenerate sites to infer the distribution of fitness effects (DFE) and the proportion of adaptive substitutions (alpha) with polyDfe v.2.0[29] using default parameters <-m C -o bfgs>. We ran the analysis independently for the two CVI islands (11 samples in Fogo and 13 in Santo Antão), and the four Moroccan clusters.

Supplementary Method 11. Evidence for ongoing multi-variate adaptation in Santo Antão

Since its collection 37 years ago[30], a single plant from CVI (Cvi-0) was studied extensively. Many mapping studies have used recombinant inbred line (RIL) populations (Cvi-0 x L*er*-0 and Cvi-0 x Col-0)[31,32], and near inbred introgression lines (NILs) of Cvi-0 into the L*er*-0 genome[33]. The island of origin of Cvi-0 was previously unknown, but we found it clusters tightly with the Espongeiro population in Santo Antão, indicating that it was collected in this region (Supplementary Fig. 1). We conducted a literature review of studies that used the Cvi-0 x L*er*-0 RILs. We identified 47 QTL-mapping studies (Fig. 6a) that mapped 129 traits that we grouped into 23 major trait-categories. These studies localized 717 QTL intervals. Based on these studies and follow-up fine mapping, we compiled a set of 135 candidate genes[4914,31–62,62–102]. In eleven cases, the actual mutation responsible for an effect on phenotype was found and validated (by

complementation tests, transgenics, sequence analyses). These variants include two large deletions, three small indels (frameshifts) and six SNPs (non-synonymous amino acid changes and truncating variants). To genotype large deletions in the natural population, we computed average coverage across 100 bp windows overlapping the deletions and flanking regions. The phenotypes affected by the functional variants range from flowering time and light signalling (FRI K232X[86], CRY2 V367M[84], GI L718F[48,88]), circadian clock regulation (ZTL P35T[88]), stomatal aperture (MPK12 G53R[92]), freezing tolerance (*CBF2* promoter deletion[103]), pathogen resistance (cPGK2 S78G[104] and *RPM1* whole gene deletion[78]), chloroplast morphology (FtsZ2-2 G441fs[87]), fructose signalling (ANAC089 S224fs[59]), and innate immunity (FLS2 N452fs[81]). In one case, a functional variant responsible for copper detoxification was identified (HMA5 N923T[56]), but it likely arose in Cvi-0 in the laboratory, since it is completely absent from the sampled natural population.

To assess the effects of the seven functional variants segregating in Santo Antão on fitness, we used forward-backward stepwise regression (i.e., sequential replacement) approach in a linear model framework using the R package caret v.6.0-86[105]. For the forward case, we started with a model with no predictors (only an intercept), iteratively added functional variant predictors, and stopped when the improvement was no longer statistically significant based on the change in root mean squared error (RMSE). For the backward case, modelling started with the full model (intercept plus all functional variants), iteratively removed the predictors that contributed the

least, and stopped when all predictors were statistically significant. Significance of models was assessed based on the root mean squared error (RMSE), by bootstrap resampling (1000 times).

To test whether the explanatory power of the seven functional variants was higher than randomly selected genomic variants, we resampled 2000 sets of seven randomly chosen variants from an LD-pruned genome (PLINK[15] command: <--indep-pairwise 50 10 0.1>) and conducted stepwise regression on each of these sets, exactly as we had done on the seven functional variants. We calculated the model R^2 to produce a null distribution and obtained an empirical *p*-value by comparing the observed R^2 value to this using the formula: $(1 + \text{sum}(s \geq s_0)) / (N + 1)$, where s is the R^2 value per draw, s_0 the observed R^2 value, and N the number of draws (https://github.com/HancockLab).

Supplementary Method 12. Trait mapping

To assess flowering time segregation in Cape Verde, we generated three inter-island F2 populations (S5-10 x F13-8 (n=488), S15-3 x F3-2 (n=636), and Cvi-0 x F9-2 (n=598)). These were grown in Bronson climatic growth chambers, with settings to match CVI conditions: 20ºC during the day and 14ºC at night, with a 12h photoperiod and 70% humidity. We scored bolting and flowering time in all F2 individuals and 12 replicates per parental line, except for Cvi-0 and F9-2, for which only four replicates were grown.

To determine whether there was transgressive segregation in each of these populations against their corresponding parental lines, we used the *DunnettTest()* function implemented in the R package *DescTools* v.0.99.37[106]. We used Fisher's method[107] to calculate a combined *p*-value across the set of crosses, using the function *fisher.method()* implemented in the R package *metaseqR* v.1.26.0[108] (https://github.com/HancockLab/CVI).

Bulked segregant analysis was done in an inter-island F2 population (S5-10 x F13-8, n=488), in which the ancestral allele FRI K232 was fixed), grown under simulated CVI conditions. Because early flowering segregated at approximately a 1:3 ratio (indicating a single recessive locus), we sampled leaf tissue from the 25% early tail of the F2 (n=108). We extracted DNA using a DNeasy Plant Mini kit (Qiagen), assessed DNA quality and quantity with Qubit and Nanodrop (Thermo Fisher Scientific), prepared a single library using NEBNext Ultra II FS DNA Library Prep Kit (New England Biolabs) and sequenced it to 50x coverage using the Illumina HiSeq3000 platform. We called variants against the TAIR10 reference assembly using a GATK pipeline[8] (https://github.com/HancockLab/CVI), retaining only biallelic variants. We identified window(s) where the median allele frequency was greater than 95% and annotated variants within candidate region(s) using SnpEff v.3.0[28].

Supplementary Method 13. *FLC* RNA quantification

We measured *FLC* expression in a representative set of eight Cape Verdean, and six Moroccan accessions. We also measured *FLC* expression in the Col-0 reference strain, as well as a modified Col-0 with a functional *FRI* introgressed (Col-0 *FRI*-Sf2, shown as Col-0 $FRI^{+}FLC^{+}$), since FRI affects *FLC* mRNA levels[109,110], and Col-0 *FRI*-Sf2 with an *FLC* knock-out (Col-0 *FRI*-Sf2 *flc-3*, shown as Col-0 $FRI^{+}FLC^{-}$)[111]. We grew three replicates of each genotype under CVI simulated conditions (12h light, 20ºC day, 14ºC night). We collected and immediately froze 2-3 rosette leaves each from 2-week-old plants and ground them with the TissueLyser II (Qiagen). We extracted RNA with TRIzol (Invitrogen) and treated 2µg with the DNA-free DNA Removal Kit (Invitrogen) for 1h at 37ºC. We generated cDNA using the Superscript II reverse transcriptase (Invitrogen) together with oligo(dT) primer for 2h at 42ºC. We assessed mRNA levels by qRT-PCR on a LightCycler 480 instrument (Roche) with the EvaGreen dye (Biotium) using the $2^{-\Delta\Delta Ct}$ method (Applied Biosystems) and *PP2A* (AT1G13320) as a reference gene. Primers used in this experiment are listed in Supplementary Table 9. Differences in *FLC* expression between genotypes were tested with the Kruskal-Wallis method implemented in the R package *agricolae* v.1.3-2[112] (https://github.com/HancockLab/CVI).

Supplementary Method 14. *FLC* complementation test

We performed genetic complementation tests for *FLC* by crossing four individuals from Fogo (each with the FLC 3X allele) to Col-0 *FRI*-Sf2 plants with and without a functional *FLC* allele (Col-0 *FRI*-Sf2, referred to as Col-0 $FRI^{+}FLC^{+}$, and Col-0 *FRI*-Sf2 *flc-3*[111], referred to as Col-0 $FRI^{+}FLC^{-}$, respectively). We also crossed the mutants (Col-0 background) to obtain a

heterozygous F1 at *FLC*. We grew four replicates of each parent and F1 per cross and scored bolting and flowering time in 12h standard greenhouse conditions.

Supplementary Method 15. Historical reconstruction of evolution of *FRI* and *FLC* loci and fit to models of adaptation

We used RELATE v1.1.4[113] to infer the genealogical trees for the derived alleles FRI 232X (Chr4:269719) and FLC 3X (Chr5:3179333). We used bcftools v1.9[114] to filter the VCF file for quality, removed non-biallelic SNPs, retained segregating sites, and filtered out missing data with the following execution <bcftools view -m2 -M2 -v snps –min-ac=1 -i ‘MIN(FMT/DP)>3 & MIN(FMT/GQ)>25 & F_MISSING=0’>. For FLC 3X, because the derived allele is fixed in Fogo, we included S1-1 from Lombo de Figueira, Santo Antão, as the outgroup. Within RELATE, we used the command RelateFileFormats (using --mode ConvertFromVcf) to convert the VCF file into haplotype and sample files. To infer the genome-wide genealogies, we first ran the command Relate (using –mode All) per chromosome and defined parameters of the recombination map (--map), mutation rate (--m), and the coalescence file (--coal) for N_e over time. The estimated mutation rate ($7x10^{-9}$) for *A. thaliana*[115] was corrected for the percentage of missing data for each region and -m set to $2.512x10^{-9}$ for FRI 232X and $2.1x10^{-9}$ for FLC 3X. We used a published recombination map[116] corrected for the estimated outcrossing rate of 5% estimated in natural populations[117] by dividing the genetic distances by 20. We then used the output to estimate coalescence rates using the script EstimatePopulationSize.sh across all 5

chromosomes with generation time set to one year, running the algorithm for 10 iterations. To obtain 95% confidence intervals for RELATE-inferred coalescence rates, we used genome-wide genealogies and coalescence rates as inputs into the COLATE package[118] (https://github.com/leospeidel/Colate), which uses a block bootstrap (100x) over genomic regions.

To infer the local genealogies for FRI 232X and FLC 3X, we ran RELATE and used the genome-wide coalescence rates (--coal) inferred previously. To produce genealogical trees for FRI 232X and FLC 3X variants with confidence intervals for the estimated ages based on 200 samples from the MCMC (derived using SampleBranchLengths.sh --format a, and using default settings), we used the script TreeViewSample.sh, with 10*N steps (N is the number of haplotypes) and 1000 burn-in iterations.

We used CLUES[119] to infer the frequency trajectory and selection coefficient for the derived FRI 232X (Chr4:269719) and FLC 3X (Chr5: 3179333) alleles. CLUES uses importance sampling over trees generated in RELATE to produce a posterior distribution of trees from which a frequency trajectory can be inferred. From the output from RELATE, we used the command <./SampleBranchLengths.sh --format b> to obtain 200 samples from the MCMC. For FRI 232X, we integrated a pseudo-ancestor individual (from our inferred CVI ancestral states) as an outgroup for the Santo Antão population. Then, we inferred genome-wide and local genealogies and conducted importance sampling in RELATE after adjusting mutation rate (-m) to 3.24×10^{-10} and 4.193×10^{-10} based on the proportion of missing data removed for the *FRI* and

FLC regions, respectively. We obtained estimates of the posterior distributions of allele frequencies over time using a recessive model (--dom 0): <inference.py --popFreq 0.7513 --tCutoff 5000 --coal relate.popsize.coal --sMax 1 --df 100 --dom 0 > for FRI 232X, and <inference.py --popFreq 0.99 --tCutoff 7000 --coal relate.popsize.coal --sMax 1 --df 100 --dom 0 > for FLC 3X (https://github.com/HancockLab/CVI). As in other analyses, we assumed one generation per year. We inferred selection coefficients jointly across two-time bins (epochs) for FRI 232X (0-2 and 2-4 kya) and three-time bins for FLC 3X (0-2, 2-4 and 4-6 kya) between the present day and the time in the past when the variants arose.

We calculated the fit to strong selection weak mutation (SSWM) and weak selection strong mutation (WSSM) models of evolution[120–122] using an estimate of the genome-wide mutational target size based on molecular studies[109,123–126] and inferences from our population genetic analyses. The logic and details can be found in the Supplementary Notes.

We conducted forward simulations in SLiM[25] under a Wright-Fisher model based on parameter estimates from the Fogo population to examine the probabilities of fixation of an adaptive variant (i.e., one that abolishes the vernalization requirement for flowering) taking into account the stochastic effects of drift. The (constant) population size was set to N=48 based on the estimate from RELATE and the selection coefficient was set to s = 0.09273 based on the estimate from CLUES under a model where the reconstructed N_e was used in RELATE/CLUES. The final number of generations depended on when the variant fixed with a maximum of 6000

generations. We simulated two genomic elements: one of size 1.5 Mbp where neutral mutations could arise and another of 1 bp where the selected variant could arise. We used three different plausible estimates for the degree of selfing (90%, 95% and 99%) based on estimates from natural population[117] and conducted 200 simulations for each case. From these, we calculated the proportion of runs where populations adapted, the proportions of potentially adaptive variants that are lost or fixed in all runs, and the times to fixation or loss.

Supplementary Note 1. Population history reconstruction

We used Chromopainter[127] to identify the closest ancestor to CVI across the genome by matching haplotypes to sequenced African and European individuals[4,128]. We found that the Moroccan High Atlas population was the closest relative for the majority of the genome (approx. 61%), followed by the North Middle Atlas population (approx. 7%) and then other Moroccan and European populations (Supplementary Fig. 4). Some of the variance visible in Supplementary Fig. 4 matching across populations may be due to the lack of a strong (close) match, so that multiple nearly equivalent distant matches can be often found. We next examined two specific large-scale loci (the chloroplast and the S-locus), where interpretation of ancestral sharing may be simpler due to the very low probability of recombination at these loci. At the chloroplast, we found that Cvi-0 clusters most closely with individuals from the South Middle Atlas population (Supplementary Fig. 4-5).

The S-locus is a well-characterized region responsible for self-incompatibility in *A. thaliana.* In the species, three deeply diverged haplogroups segregate (A, B, C) as well as an ancient recombinant (A/C) haplotype[129–131]. Due to the deep divergence between the major haplogroups, recombination between these is suppressed and thus exceedingly rare in this region[132] so that matching to major haplogroups is clear. We classified S-locus haplogroups in all CVI samples following [4] and found that they all carried haplogroup B (Supplementary Fig. 4). In the continental sample, this haplogroup is present only in three samples and only in the northernmost Moroccan population in the Rif Mountains[4,133].

Taken together, these patterns show that while Morocco is the continental population genetically closest to CVI, there is no single Moroccan sample or population that is consistently closest to CVI across the genome. Instead, our findings suggest that CVI was colonized by a 'ghost' population that is not represented well by any modern-day sampled population.

Previously, based on the timing of coalescence events, we inferred that Moroccan populations expanded and contracted over time[4]. This could have led to loss and/or re-sorting of lineages among populations and could help to explain why we were unable to identify a single best representative of the colonizing population[4].

Next, we narrowed down the CVI colonization time by obtaining an upper bound based on the minimum coalescence time between CVI and Morocco and a lower bound based on the maximum coalescence time within the CVI clade.

To estimate the split time between Moroccan and CVI populations, and therefore the upper bound of colonization time, we calculated the relative ratio of between-group coalescence events to within-group coalescences (i.e., the cross-coalescence rate (CCR) statistic) implemented in MSMC[16,134]. Given that we are unable to identify the closest Moroccan population, we expected this analysis to overestimate the divergence time from the actual continental ancestor. The CVI population exhibited initial divergence from all present-day Moroccan populations at approximately 40-60 kya (Supplementary Fig. 7), which we interpret to represent the split time between the present-day Moroccan population and the 'ghost' ancestor of the CVI populations.

CCR between CVI and High Atlas shows a somewhat different pattern compared to other Moroccan populations. The trajectory of the statistic does not monotonically decay as would be expected under a simple split model but rather inflects and reaches a local maximum between 10 and 20 kya (Supplementary Fig. 7). As a result, the 0.25 - 0.75 CCR quantiles for the CVI-High Atlas split are consistent with a wide range of split times from 60 kya until as recently as 10 kya. This inflection could potentially be explained by a complex relationship between the ancestors of the Moroccan and CVI lineages, i.e., secondary contact between the ancestors of the 'ghost' and

the High Atlas populations. This resulted in the presence of some haplotypes across the High Atlas genomes that represent the population that originally colonized CVI.

Although we were unable to identify a close outgroup population to CVI, we can use information about the coalescences within CVI to obtain a lower bound on the colonization time. We examined historical coalescence events in Santo Antão, Fogo and between the two islands based on haplotype coalescences. Coalescence rates spike around 10 kya (Supplementary Fig. 7) and decline sharply in Santo Antão starting at approximately 7 kya, when we infer population structure begins to develop within this island (Supplementary Fig. 8d). In agreement with this time estimate, and based on the density of mutations, 95% of genomic windows between islands (0.1 Mb, non-overlapping) coalesced by 7.1 kya (Supplementary Fig. 8b). Then, at approximately 4-5 kya, there is a strong signature of reduced coalescence between islands, consistent with a split at this time (Supplementary Fig. 8d).

The long gap between the coalescence of lineages within CVI (5-7 kya) and the coalescence times between present-day Moroccan and CVI populations (40-60 kya) is consistent with a model in which a now extinct or unsampled ‘ghost’ population was the actual founding population of CVI. Based on our analyses, we hypothesize that this population split from the present-day Moroccan population approximately 35-50 kya (Supplementary Fig. 7) and that island colonization likely occurred approximately 7 kya, when population structure becomes apparent in CVI (Supplementary Fig. 7).

To further explore the colonization dynamics and to assess evidence for colonization by a ‘ghost’ population in different time frames, we compared the distribution of ages of genomic windows (haplotypes) to those from simulations of a ‘ghost’-CVI split at different time points (5, 10, 20, 30, 40 and 50 kya). These simulations were conducted under the assumption that variable mutation rate and purifying selection reduce diversity by the same extent as divergence (the same rationale as in the HKA test) and allow for testing for secondary contact between the ancestors of the Moroccan and ‘ghost’ populations. In that way, we were able to capture the genomic variance in the combined $N_e\mu$ parameter by scaling the simulations to diversity in Morocco across genomic regions. This approach was based on the logic that the inflection in the MSMC-CCR plot (Supplementary Fig. 7) could be due to secondary contact between the ‘ghost’ population and the Moroccan population (i.e., gene flow back into the Moroccan population from the ‘ghost’). In this case, we could use the distribution of window ages (inferred based on the density of SNP variants) to estimate the timing of the secondary contact event. This timing inference would better represent the split between CVI and the ‘ghost’, although it is still likely to be an overestimate. We found that the cumulative tail of recent coalescence times in observed data fits best with a 10 ky old split and upper bound of colonization time (Supplementary Fig. 7).

The site frequency spectrum provides complementary (largely independent) information from signatures of haplotype coalescences. We ran dadi to infer the split time between Morocco and the ancestor of the CVI population. We used five demographic models including 1) a simple

split model, 2) a model that included an exponential population size change in CVI after the split, 3) an isolation-with-migration model, 4) a model that included a bottleneck in CVI after the split, followed by an instantaneous size change, and 5) a model that included bottlenecks in both the CVI and Moroccan populations after the split and a subsequent size change in CVI. Details of the model parameters are shown in Supplementary Fig. 7. The best performing model (based on AIC) is the two-bottleneck model. This model includes a bottleneck in CVI after the split, followed by an instantaneous population increase (Supplementary Fig. 7). Similar to the haplotype coalescence analysis, the parameter estimates under this JSFS-based model capture the signal of an early split between the ancestor of the CVI colonist ('ghost' population) and the ancestor of the current Moroccan population, placing this split time at 49.7 kya, with a 43.5 kya bottleneck. This scenario fits well with the inferences from haplotype coalescence times (Supplementary Fig. 7) where the long-term effective population size of the 'ghost' population was small in the interval between the split of the parental populations and expansion within CVI. In the JSFS-based model, the time when the CVI population is inferred to increase in size (6 kya) is consistent with an expansion beginning sometime after the colonization of the islands (Supplementary Fig. 7d-e). Neither the haplotype coalescence approach nor the JSFS approach between Morocco and CVI can reveal specific information about the propagule size to CVI due to confounding with the 'ghost' population. However, the very low number of shared variants between present-day Moroccan populations and CVI (0.1%) suggests that the colonizing population was depleted of variation and that current trait variation in the islands occurred via new variants.

Next, we wanted to determine the order of island colonization. Isolation by distance leads to a reduction in variation with distance from the starting population and to a pattern in which a proportion of derived variants that segregate in one (parent) population will be fixed in the child population[135,136]. Therefore, we focused on the subset of variants that are segregating in one island and fixed derived in the other to infer colonization order. First, we examined our power for this approach using simulations. Here, we found that as long as the time between the colonization of the first and second island, or the size of the island colonized first is large enough (larger than approximately 500 individuals at the split), the island colonized first will have a lower proportion of fixed mutations that segregate in the other island, independent of the number of colonizers. In the observed data, the Fogo population has a higher proportion of fixed mutations that segregate in Santo Antão compared to the converse (a positive statistic in Supplementary Fig. 8c), supporting initial colonization of Santo Antão, followed by Fogo (from Santo Antão). When we fit the statistic estimated from data to forward simulations, we inferred that population size in Santo Antão grew quite slowly from colonization until the split from Fogo, with a final size of only about 1000 plants when Fogo was colonized.

Further, we used dadi[17] to fit three-population models to the observed join site frequency spectra. The models were simple 3-populations splits with constant population sizes, but they differed in which island was colonized first. In the first model, Santo Antão was colonized from Morocco, and later Fogo was colonized from Santo Antão. In the second model, Fogo was

colonized from Morocco, and later Santo Antão was colonized from Fogo. The model that best fit observed data (lowest AIC, 612 vs 628; highest likelihood, -301.3 vs -309.1) was the one in which Santo Antão was colonized first from Morocco, and Fogo was colonized later from Santo Antão.

Next we inferred the split time between islands and other aspects of historical population dynamics using the JSFS (dadi[17]). We estimated parameters under a range of models (simple split, exponential, isolation with migration, bottleneck) and compared model AICs to identify the best fitting model of the historical dynamics of the Fogo population. We found that the best model was one in which the island colonization event was accompanied by a bottleneck lasting 930 years. The population size (N_e) during the bottleneck period was approximately 400 individuals (Supplementary Fig. 8a, Supplementary Table 3). We did not attempt to estimate the number of founders separately from the bottleneck size because diffusion models generally do not perform well, such as dadi, generally does not perform well to infer the N_e before a bottleneck[17].

The estimated split time is in rough agreement with a simpler estimate based on the distribution of pairwise differences across windows within and between islands (Supplementary Fig. 8b). We calculated the mean pairwise differences among CVI individuals across 100 kb windows of the genome and found that coalescence time between islands was centred around 4.5 kya (mean: 4.6 kya median: 4.5 kya) and that 95% of the windows coalesced by 7.1 kya. The

complete distribution of pairwise differences is shown in Supplementary Fig. 8b. Variation within Santo Antão was centred around 3.0 kya (mean: 3.0 kya; median: 3.0 kya) with 95% of windows coalescing by 4.8 kya and in Fogo around 2.4 kya (mean: 2.5; median: 2.4) with 95% of windows coalescing by 3.9 kya.

The bottleneck duration (T_s) agrees with a simple calculation based on the proportion of fixed variants in Fogo. The Fogo population carries 135 fixed derived mutations out of a total of 23 Mbp of sequence. Using T_s = number of fixed mutations/(μ*L), we can estimate about 840 generations, in which Fogo remained a single connected population (about 840 years) after colonization before structure built up preventing mutations from fixing in the island. The same calculation based on intergenic sites only, results in a very similar bottleneck duration (36 fixed derived intergenic mutations out of 4.8 Mbp: about 1070 years).

The complete lack of population structure for approximately 870-1070 years for the Fogo population is striking. Seed-dispersed plant populations tend to be highly structured[137] including *A. thaliana* populations[138–141]. However, we find no evidence of structure accumulating in the long post-colonization bottleneck period in Fogo. This implies that the nascent population was poorly adapted to its new environment and therefore severely limited in size during this waiting period until one or more necessary mutations arose that increased fitness and allowed the population to expand.

Secondary migration events after initial colonization are unlikely. Allowing for migration (gene flow) between the Moroccan and 'ghost' populations after the split led to a poor model fit (based on AIC; Supplementary Fig. 7), implying a lack of migration after the split. This is not surprising given that multiple independent migration events into CVI would likely lead to much higher genetic variation than we observe in the archipelago. The average pairwise differences between two Moroccan individuals is 82.4-fold higher relative to the average pairwise differences within CVI (θ_π (Morocco) = 5.38x10^{-3}; θ_π (CVI) = 6.53x10^{-5}). Even within a single Moroccan region, the average pairwise differences is 54.2-fold higher than in CVI (on average θ_π (Moroccan regions) = 3.54x10^{-3}; Supplementary Table 1). Further, we observed extremely low sharing of variation between CVI and Morocco (0.1%). Based on this, it is difficult to imagine a scenario in which multiple independent dispersal events could have contributed to the present CVI populations.

Similarly, given the almost complete lack of shared variants genome-wide between Santo Antão and Fogo (0.6%), it is highly unlikely there was any subsequent migration after the initial colonization of Fogo. In further support of this assertion, a single chloroplast haplotype is fixed in CVI, with only 18 variants segregating there. Similar to clustering from genomic variation, a chloroplast network clearly separates the two islands (Supplementary Fig. 6). Consistent with the lack of secondary migration between islands, demographic inference with dadi finds the best

support for a model with no migration (Supplementary Fig. 8c-d). Further, given the inferred small initial population size in Fogo, a secondary migration event from Santo Antão would likely result in much higher shared variation between islands than observed.

Supplementary Note 2. Population history within islands

Trajectories of coalescence rates within Santo Antão and Fogo (MSMC) as well as matching to patterns of polymorphism in simulations (Supplementary Fig. 8d) imply that Santo Antão was colonized first and Fogo second from Santo Antão. Trajectories of coalescence rates over time for individual Santo Antão populations and inferred split times with MSMC-CCR show that the Cova de Paúl population best represents the early colonists (Supplementary Fig. 8d). Based on CCR analysis, we estimated that Cova de Paúl split from Lombo de Figueira at approximately 7 kya. At this time, the coalescence (MSMC) trajectory for Santo Antão enters a period of intense reduction (Supplementary Fig. 7b), which likely corresponds to the formation of population structure as *Arabidopsis* expanded its range. The most recent population splits are between Espongeiro and Pico da Cruz. In Fogo, the more arid island, we found evidence for a bottleneck that lasted approximately 930 years after colonization (Supplementary Fig. 8a, Supplementary Table 3). Once the population did begin to expand in Fogo, it dispersed to three regions (Monte Velha, Inferno and Lava) and structure developed among these.

Supplementary Note 3. Niche modelling to predict suitability of Cape Verde habitat based on Moroccan distribution

The variables in the final Maxent model were the length of the growing season, isothermality, maximum temperature of the warmest month, minimum temperature of the coldest month, temperature annual range, mean temperature of the wettest quarter and precipitation seasonality. When we projected the Moroccan niche model onto the Cape Verde archipelago, we found that there was no habitat predicted to be suitable for colonization from Morocco. As is generally the case in niche modelling, correlations between environmental variables across the range result in alternative possible models. The model we present uses a set of variables chosen based on ecological and biological relevance. However, predicted suitability of CVI habitat for Moroccan accessions did not change across different variable selection regimes: none predicted suitable habitat for Moroccan *A. thaliana* establishment in CVI. This is likely due to the fact that the climate variable values for CVI lie outside or nearly outside the distribution of most Moroccan climatic variables. As a result, the predicted (dis-)similarity may be more informative, which shows that much of Santo Antão and a band around the highest altitude Bordeira region of Fogo has the highest predicted similarity. These most similar regions encompass locations where we found populations of *Arabidopsis* in CVI.

Supplementary Note 4. Evidence of positive selection in CVI

We computed the relative rate of fixation of 0-fold non-synonymous to 4-fold synonymous variants (d_{sel}/d_{neu}) for the Moroccan and CVI lineages. In the absence of selection $d_{sel}/d_{neu}=1$, as non-synonymous and synonymous mutation have the same probability to arise and fix. Purifying selection reduces the probability of fixation of non-synonymous mutations resulting in $0 \leq d_{sel}/d_{neu} < 1$. A relaxation of purifying selection will move the d_{sel}/d_{neu} ratio from $0 \leq d_{sel}/d_{neu} < 1$ towards neutrality, $d_{sel}/d_{neu}=1$. Positive selection is in principle the only force that can result in higher substitution rates of functional compared to neutral mutations, resulting in $d_{sel}/d_{neu} > 1$. However, multiple forces are likely to be acting at any one time across loci in the genome, so that the observed d_{sel}/d_{neu} is expected to represent a composite of the neutral evolution, purifying selection and adaptive evolution. Therefore, $d_{sel}/d_{neu} > 1$ implies that there are many advantageous mutations fixed on the branch leading to the clade.

Consistent with the large-scale effect of purifying selection in continental *A. thaliana*, the d_{sel}/d_{neu} ratio in Morocco, polarized to *A. lyrata*, is 0.18 (Fig. 5a). For Santo Antão and Fogo, we analysed the long branch of divergence from the continents, on which mutations are found fixed derived in CVI as a whole, as well as the two short branches separating the two islands, where mutations are fixed derived in one or the other island. The long branch of divergence mainly represents the continental history in the 'ghost' population but it is also confounded with the early history in CVI after colonization. In this case, we obtained a d_{sel}/d_{neu} ratio of 0.276, slightly higher than the Moroccan population. Then we examined the short branches separating the two

islands, which correspond to the past 4-10 ky of evolution in isolation within Santo Antão and Fogo. In this case the genome-wide d_{sel}/d_{neu} ratios (Santo Antão: d_{sel}/d_{neu}=2.2, Fogo: d_{sel}/d_{neu}=1.7) are much higher than for the Moroccan population and also higher than unity, consistent with strong positive selection acting across the genomes of the island populations. Due to the very shallow history within each island, few mutations had the time to arise and fix in each functional category; as a consequence, confidence intervals around these estimates are necessarily high, but nonetheless consistent with exceptionally high d_{sel}/d_{neu} values in CVI. To further investigate these patterns, we used the software polyDfe[29] to estimate the statistic alpha, the proportion of non-synonymous substitutions driven by positive selection or linked to positively selected alleles. Consistent with the high d_{sel}/d_{neu} ratio, we estimate that in Santo Antão 70.7% and in Fogo 62.8% of non-synonymous substitutions were driven to fixation by positive selection or by linkage to positively selected alleles.

Additionally, we estimated the distribution of fitness effects (DFE) of variants segregating in the CVI and Moroccan populations with polyDfe[29]. The DFE in the two Cape Verde islands are enriched both in neutral and slightly deleterious variants (-1, 0 category), as well as in variants of large positive effects on fitness (all positive categories) compared to Moroccan populations (Fig. 5b). This is consistent with a reduction in efficiency of purifying selection relative to the continent combined with strong positive selection in response to the novel CVI environment. The two islands differ somewhat in the inferred parameters of positive selection. In Santo Antão, the percentage of variants estimated to have a positive effect on fitness (p_b) was 2.9%, with an

average effect of $S_b = 54.7$. In Fogo, the percentage of beneficial variants was greater, $p_b = 19.7\%$, but with a smaller average inferred effect on fitness, $S_b = 6.8$.

Supplementary Note 5. Parallel adaptation by reduced time to flowering

We mapped flowering time in Santo Antão using GWAS, with a linear mixed model (LMM) that controls for population stratification[142]. We used the median per genotype across replicates as phenotype, since no block effect was detected in the simulated CVI conditions experiment (Supplementary Fig. 9). All typed variants in the genome explained 99.997% of the observed phenotypic variance (PVE; also known as 'chip heritability' or 'SNP heritability') and we identified one clear genome-wide significant peak on top of chromosome 4. This peak included FRIGIDA (*FRI*, AT4G00650; likelihood ratio test, p-value = $5.468\text{x}10^{-35}$), a major flowering time determining gene. The nonsense mutation FRI K232X in Cvi-0 truncates the protein and was previously shown to strongly reduce flowering time[86]. Adding FRI K232X to our model as a covariate allowed us to quantify the association with the phenotype. We found that when FRI K232X was added to the model, the percentage of phenotypic variance explained by all remaining markers decreased to 53.59%, with an estimated effect size for the covariate of -35.27 ± 1.50 days. This means that this single SNP is able to reduce flowering time in this population in about 35 days and explain 46.41% of the phenotypic variance. Concordant with this estimate, in the natural population, FRI 232X is associated with a decrease in flowering time of 34 days (MWW test, $W = 7$, p-value < $2.2\text{x}10^{-16}$), and a 140-fold increase in seed number (+387 seeds;

MWW test, W = 4541, *p*-value = 7.179×10^{-14}; Fig. 6e). In the Col-0 background, under CVI conditions, a non-functional *FRI* allele was responsible for a decrease in flowering time of 27 days (MWW test, W = 0, *p*-value = 0.00384) and an increase in fitness of 669 seeds (MWW test, W = 37.5, *p*-value = 0.008856; Fig. 6e).

In Fogo, we did not find any statistically significant association in GWAS for flowering time (Supplementary Fig. 11), suggesting that the genetic variant(s) underlying the uniformly reduced flowering time were fixed or at high frequency (mean=28.72 days, SD=3.76). Taking this into consideration, we scored flowering time in an inter-island F2 population in which the ancestral allele FRI K232 was fixed. We observed early flowering individuals segregating at approximately 1:3 ratio, indicating that a single recessive locus is causing the early flowering phenotype. After bulking and sequencing the early tail of this distribution (n=108), we identified a single region on chromosome 5 between 2 Mbp and 3.3 Mbp where the frequency of the Fogo alleles was greater than 95% in the sequenced pool. Among the 33 variants in this region (Supplementary Data 6), 14 are at a frequency greater than 90% in the natural population and therefore they are stronger candidates to explain the uniform early flowering observed in Fogo. Of these 14, two are predicted by SnpEff[28] to have moderate impact, and one to have high impact.

The two moderate impact variants affect AT5G09930, an ABC transporter protein, causing a missense mutation (V193F), and AT5G07520, a glycine-rich protein expressed only in flowers during a specific developmental stage (flower stage 12), with an 11 amino acid deletion (A202_A213del). The high impact variant is predicted as causing a premature stop codon in AT5G10140 (R3X). AT5G10140 is FLOWERING LOCUS C (*FLC*), a MADS-box protein central to the flowering time pathway. *FLC* is regulated by FRI and vernalization, contributes to temperature compensation of the circadian clock, and acts as a repressor of floral transition[143,144]. Due to its central function in flowering time, *FLC* is the best candidate for the early flowering observed in Fogo.

Although the flowering time gene *FLC* contains a premature truncation variant at the third amino acid fixed in Fogo, the gene could be functional, e.g., due to an alternative start codon or transcriptional read-through. To functionally characterize the nonsense mutation in *FLC* in Fogo, we quantified *FLC* mRNA levels in the natural population, and compared them to *FLC* mRNA levels in Santo Antão, Morocco, the Col-0 reference strain, and Col-0 *FRI*-Sf2 (functional *FRI* in Col-0 background) with and without a functional FLC[111] (noted as $FRI^{+}FLC^{+}$ and $FRI^{+}FLC^{-}$, respectively; Supplementary Fig. 12a).

Compared to $FRI^{+}FLC^{+}$, transcript levels of *FLC* were reduced in Fogo individuals (Kruskal-Wallis, p-value < $1\text{x}10^{-4}$), as expected if FLC R3X results in non-functional FLC.

Similarly, *FLC* transcription was reduced in $FRI^{+}FLC^{-}$ and Col-0 wild-type (non-functional FRI and functional FLC) compared to $FRI^{+}FLC^{+}$ (Kruskal-Wallis, *p*-value < $1x10^{-4}$), which was consistent with previous findings[109]. In contrast, all accessions from Santo Antão showed high levels of *FLC* expression (comparable to $FRI^{+}FLC^{+}$; Kruskal-Wallis, *p*-value > 0.6783). This result is in agreement with previous work showing a higher baseline expression of *FLC* in Cvi-0[86,109]. Within Santo Antão, the single sample tested with a functional FRI (S5-10) had higher *FLC* expression compared to the other samples from Santo Antão (Kruskal-Wallis, *p*-values: Cvi-0=0.0006; S1-1=0.0039; S15-3=0.0008), consistent with an effect of *FRI* on *FLC* expression even in the CVI genetic background[86,109]. In Moroccan accessions, we observed high levels of *FLC* expression across populations, with much larger variation.

The two loci, *FRI* and *FLC*, explain the differences in bolting time in the tested accessions (Supplementary Figure 12b). In the Col-0 background, when both loci have functional alleles ($FRI^{+}FLC^{+}$), the plants bolted later than when either of the loci had one non-functional allele (non-functional FRI in Col-0 and non-functional FLC in $FRI^{+}FLC^{-}$; Kruskal-Wallis, *p*-value = 0.0047 and *p*-value = 0.0002, respectively). In Santo Antão, all individuals bolted early, comparable to Col-0 (Kruskal-Wallis, for S1-1, S15-3, Cvi-0: *p*-value = 1) and $FRI^{+}FLC^{-}$ (Kruskal-Wallis, for S15-3, Cvi-0: *p*-value = 1, for S1-1: *p*-value = 0.2376), as all accessions carry a non-functional FRI. The exception was S5-10, which bolted later (Kruskal-Wallis, *p*-values: Cvi-0=0.0005; S1-1=0.5811; S15-3=0.0026) due to a functional *FRI* allele. This accession also showed higher levels of *FLC* expression, consistent with a role of regulation of

FLC mRNA levels by *FRI*[86,109]. In Fogo, the non-functional FLC present in all accessions is consistent with the early bolting and the low levels of *FLC* mRNA, both comparable to the non-functional FLC mutant in the Col-0 background (Kruskal-Wallis, for F10-1-3, F13-8, F9-2: *p*-value = 1, for F3-2: *p*-value = 0.2014). In Morocco, the levels of *FLC* mRNA did not completely explain the bolting time, suggesting that other loci may influence this trait in this population.

To further test whether *FLC* is responsible for the early flowering phenotype in Fogo, we conducted a genetic complementation test, by crossing 4 individuals from Fogo (all with a potential non-functional *FLC* allele) to Col-0 *FRI*-Sf2 with and without a functional FLC (noted as $FRI^{+}FLC^{+}$ and $FRI^{+}FLC^{-}$ [(111)], respectively; Supplementary Fig. 12).

If the allele in Fogo was non-functional, when crossed to $FRI^{+}FLC^{-}$, all F1 individuals would flower as early as $FRI^{+}FLC^{-}$, as they would carry non-functional *FLC* alleles from both parents. In this case, F1 individuals homozygous for the null-allele at *FLC* would also flower earlier than heterozygous individuals (F1 from the cross between $FRI^{+}FLC^{+}$ and $FRI^{+}FLC^{-}$*)*, given that the functional *FLC* allele should be dominant[109,111]. On the other hand, if the *FLC* allele in Fogo was functional, the F1 individuals from this cross would have one functional allele and flower later than $FRI^{+}FLC^{-}$*,* and similar to the heterozygous individuals (F1 from the cross between $FRI^{+}FLC^{+}$ and $FRI^{+}FLC^{-}$). In the inverse cross – between Fogo and $FRI^{+}FLC^{+}$ – if the Fogo

allele is non-functional, we expect the F1 to be heterozygous and flower as late as *FRI*$^{+}$*FLC*$^{+}$ and similarly to the F1 resultant from the cross between *FRI*$^{+}$*FLC*$^{+}$ and *FRI*$^{+}$*FLC*$^{-}$.

For the crosses between Fogo individuals and *FRI*$^{+}$*FLC*$^{-}$, we found that all F1 individuals flowered as early as the parental line *FRI*$^{+}$*FLC*$^{-}$ (MWW test, W = 42, *p*-value = 0.3611), much earlier than the *FRI*$^{+}$*FLC*$^{+}$ (MWW test, W = 0, *p*-value = 0.00248) and the F1 heterozygous between *FRI*$^{+}$*FLC*$^{+}$ and *FRI*$^{+}$*FLC*$^{-}$ (MWW test, W = 4, *p*-value = 0.0002342). On the other hand, all the F1 individuals from the cross between Fogo and *FRI*$^{+}$*FLC*$^{+}$ flowered as late as the FLC functional parental line *FRI*$^{+}$*FLC*$^{+}$ (MWW test, W = 34, *p*-value = 0.8814), much later than the Fogo parents (MWW test, W = 256, *p*-value = 1.301×10^{-6}) and also later than the F1 between Fogo and *FRI*$^{+}$*FLC*$^{-}$ (MWW test, W = 116.5, *p*-value = 0.001227). These results together suggest that the *FLC* 3X allele found in the natural population in Fogo is indeed non-functional.

Next, we inferred the coalescent genealogies of FRI 232X and FLC 3X using RELATE[113]. The coalescent tree for FRI 232X is bifurcated rather than hierarchical at the time when the variant is estimated to have arisen, indicating that some branches were likely lost (extinct) or unsampled in the modern populations. The time of the bifurcation is estimated at approximately 2.9 kya, with 95% CI estimated from 200 samples from the MCMC approximately 2.14 kya – 3.74 kya. The tMRCA, which represents the lower bound on the age, is 2.14 kya (95% CI: 1.62-2.72 kya). Based on the coalescent reconstruction for FLC 3X and 200 sampled trees, the allele

arose between the tMRCA at 3.3 kya (95% CI: 2.82-3.96 kya) and the split from the outgroup at 4.72 kya (95% CI: 3.56-6.66 kya).

We inferred the frequency trajectories for FRI 232X and FLC 3X using CLUES[119], which uses importance sampling over trees generated in RELATE to infer the frequency trajectories and selection coefficients for the functional variants. For each variant, we defined time bins (epochs) between the present and the emergence of the allele (based on the inferred age of the allele in RELATE when approximately 97.5% of the coalescent trees for the region support the existence of the allele). For FRI 232X, these epochs were 0-2 kya and 2-4 kya. For this variant, we found that s was maximized in the epoch 2-4 kya years ago, with a selection coefficient of 4.56% (Supplementary Table 6). For FLC 3X, these epochs were 0-2 kya, 2-4 kya, and 4-6 kya, with the inferred selection coefficient maximized 4-6 kya with s = 9.27% (Supplementary Table 7).

Supplementary Note 6. Assessing the fit of adaptation in CVI to models of selection

Theory predicts that when mutational input is low and selection is strong (SSWM regime), the first steps of adaptation are likely to occur through large effect mutations, whereas when mutational input is high and selection is weak (WSSM regime), adaptation is likely to occur

through more, smaller effect variants[120–122,145–148]. We examine the CVI case in the context of SSWM versus WSSM regimes. First, we approximate the genome-wide mutation rate for the adaptive phenotype (very early flowering through loss of vernalization) and then we apply the inferences we made about population history and selection coefficients to examine the fit of adaptation in each of the two Cape Verde islands to these two models.

The SSWM model is expected to hold when the total number of new mutations that enter a diploid population each generation is small such that $4NU_b \ll 1$ (where N is population size and U_b is the genome-wide beneficial mutation rate for the focal trait) and when selection is strong $4Ns >> 1$. In this scenario, single new beneficial mutations arise and overcome genetic drift ($s >> 1/4N$) and subsequently rise in frequency without interference (by linkage or epistasis) from beneficial alleles at other loci[120–122]. Note that small population size (as in a founder population) plays a double role. On one hand, it enables SSWM type adaptation by restricting the mutation input; on the other hand, it can inhibit adaptation altogether unless selection is strong. The combination of small population size and strong selection is expected to result in a "selective-sweep type" architecture of adaptation[149] where one or few variants with large effects underlie the adaptive phenotype. Alternatively, adaptation in the WSSM regime occurs through small frequency shifts at a large number of alleles with small individual effect. Theory shows that $4NU_b = 1$ is indeed the threshold that separates the sweep-like from highly polygenic architectures[149,150].

Time to flowering has been studied extensively in *A. thaliana* and much is known about its molecular basis. We used available molecular and functional analyses of major flowering time loci to produce rough approximations of U_b. First, we reviewed the literature to identify the loci and mutational effects that lead to effects on flowering time. Many genes can contribute to variation in flowering time (at least 174 genes are thought to be involved in the flowering time pathway (https://www.mpipz.mpg.de/14637/Arabidopsis_flowering_genes [151–154]). However, very few of these cause large or even moderate changes in flowering time. A more relevant (specific) phenotype in this case is the loss of the vernalization requirement (i.e., cold period needed to induce flowering), which results in a large reduction in flowering time. The loci most often implicated in this trait are *FRI* and *FLC*, both of which are essential for vernalization response. Loss of function of either of these genes results in loss of the vernalization requirement for flowering. When diverse rapid flowering accessions were examined, 85% turned out to have clear evidence of functional mutations in *FRI*, *FLC* or both[109,152]. In the absence of a vernalization treatment, complete loss of function of these genes results in a reduction of flowering time of approximately 35 days relative to wild type (Fig. 7e). In nature, variation in *FRI* occurs primarily by loss of function mutations in coding regions[109,123], while most putative functional variation in *FLC* is found in the first intron[124,125], which contains a well-characterized regulatory element[126].

Based on this information about the genetic basis of large effect changes in flowering time (vernalization), we can roughly estimate U_b, the genome-wide per individual mutation rate of beneficial mutations that act through the focal phenotype (the number of nucleotides whose changes would cause a large shift in phenotype).

First, we focus on loss of function mutations due to SNPs in coding regions of the genes involved in complete loss of vernalization (*FRI* and *FLC*). For these, the total length of the two genes is 805 amino acids. We estimate that on average three out of 64 mutations could lead to a premature stop codon. This results in a mutational target size for loss of function through SNPs in coding regions of 805*3/64. We assume a mutation rate by SNP changes of $7.1x10^{-9}$ [(115)]. The rate of introduction of loss of function mutations by premature stop codons per individual and per generation is therefore:

$U_{b\ (coding\_SNPs)} = 805 \text{ x } 3/64 \text{ x } 7.1x10^{-9} = 2.68 \text{ x } 10^{-7}$

We can additionally account for mutation by indels, using the mutation rate estimated from mutation accumulation lines. We used the per base mutation rate estimated for 1-3 bp long deletions, $4.0x10^{-10}$ [(115)]. The mutational target size is then 805 amino acids * 3 nucleotides/amino acid. The rate of introduction of indel mutations in the coding region is therefore:

$U_{b(coding\_indels)} = 805 \text{ x } 3 \text{ x } 4x10^{-10} = 9.66 \text{ x } 10^{-7}$

Combining the probability of mutation by SNPs or indels in coding regions gives:

$U_{b(coding)} = 2.68x10^{-7} + 9.66x10^{-7} = 1.23x10^{-6}$

Regulatory mutations in *FLC* and *FRI* also have the potential to impact flowering time. To include the possibility of regulatory mutations in our estimate of U_b, we estimated the probability that a mutation has a major regulatory effect based on the literature. Regulatory elements can include promoter regions as well as conserved non-coding elements upstream, downstream or within introns. For *FRI* and *FLC* these are well-studied. Core promoters are on average approximately 75 bp in *A. thaliana* and are tightly packed with regulatory elements[124]. Given a moderate level of nucleotide-level functional redundancy, we assumed that one in 10 possible changes in the core promoter could have major functional effects. In addition, we assumed that larger 5' regions with interspersed transcription factor binding sites would add 300 bp to the core[155]. In these regions regulatory motifs are generally more dispersed and redundancy is likely to be rather high at the nucleotide level. We assumed that on average the probability of a mutation resulting in a strong functional effect in these regions would be one in 50. In *FLC* there is evidence that motifs exist within a 'vernalization response element' in the first intron (289 bp) that are crucial to function[126]. Here, we assumed that one in 20 mutations may result in large changes in flowering time.

While we attempted to come to a meaningful approximation of the number of variants (mutational target size) that might have *major* effects on regulatory function of *FLC* or *FRI*,

assumptions about this mutational target size are necessarily less certain. However, the final estimate would not change much if other biologically informed estimates were used.

$U_{b(regulatory\ FRI/FLC\_SNPs)} = (150 \times 0.1 + 600 \times 0.02 + 289 \times 0.05) \times 7.1 \times 10^{-9} = 2.94 \times 10^{-7}$

And including indels in regulatory regions gives us:

$U_{b(regulatoryFRI/FLC\_indels)} = (41.45) \times 4 * 10^{-10} = 1.66 \times 10^{-8}$

So that the combined probability of a major effect mutation in regulatory regions by SNPs or indels is:

$U_{b(regulatory)} = 2.94 \times 10^{-7} + 1.66 \times 10^{-8} = 3.11 \times 10^{-7}$

And the combined probability of any mutation with a major effect on the vernalization requirement is:

$U_b = 1.23 \times 10^{-6} + 3.11 \times 10^{-7} = 1.54 \times 10^{-6}$

Using the estimate for U_b and an assumption about past population size, we can infer the waiting time for a mutation in the natural population. Then, using our inference about selection coefficients from the allele frequency trajectory in each island, we can assess how each estimate fits with SSWM and WSSM models. We initially focus on Fogo because the population history there is simpler and resolved with higher certainty. Then we turn to Santo Antão, and make some approximations and an estimate for that population.

For N, we used the estimate of N_e from RELATE/COLATE in the Fogo population (N_e=48 individuals at colonization). With that, the per generation population mutation rate for a strong effect functional variant in *FRI* or *FLC* would be

$$\Theta_{(FRI/FLC)} = 4N_eU_b = 4 \text{ x } 48 \text{ x } 1.54\text{x}10^{-6} = 2.95\text{x}10^{-4} << 1.0$$

There is of course uncertainty in this result from our rough estimate of both U_b and N_e. However, as our derivation leaves considerable room for error of almost four orders of magnitude, the conclusion that adaptation of our focal trait (loss of vernalization requirement) is mutation limited ($\Theta_{(FRI/FLC)} \ll 1$) appears to be highly plausible. Our estimate corresponds to an expected waiting time for the occurrence of any mutation that abolishes function of the genes of $1/\Theta$ = 3775 generations. If we break this down into coding and non-coding we find that the estimated waiting time for a coding change specifically is ($1/4N_eU_{b(coding\_FLC/FRI)}$) = 4727 generations and the waiting time for loss or major reduction of function through a regulatory change specifically is estimated at ($1/4N_eU_{b(regulatory\_FLC/FRI)}$) = 18,694 generations. Based on this, if such a variant was required to escape eventual extinction, extinction risk would be very high. Further, given the much lower non-coding adaptive mutation rate, an adaptive mutation from variation in the coding region would be much more likely in this case.

Under the SSWM model, strong selection is required in order to escape drift. Here, we apply a selection coefficient based on the reconstructed frequency trajectory of FLC 3X in the time just after it is estimated to have arisen (s = 0.0927). If FLC 3X resulted in a population size increase,

this estimate would be highly conservative because the population size change is used as the null model here. Recall that SSWM is expected to hold when $4NU_b \ll 1$ and $s >> 1/4N$. Even with the conservative estimate of s, we find that this is well above $1/4N_e$: $s = 0.0927$ and $1/4N_e = 5.21x10^{-3}$. This implies that a new mutation that obliterates the vernalization requirement in a Fogo-like environment would tend to escape drift. We conclude that the scenario is consistent with the SSWM regime, where adaptation relies on sweeps of large-effect alleles[120,147,149,150].

Of course, variance on the estimates of the time for a variant to arise and fix in the population would be high due to the stochastic nature of mutation and the uncertainty of establishment. We conducted simulations under a model designed to fit the Fogo population to quantify the probability of fixation under a constant-size Wright-Fisher model with three plausible estimates of the selfing coefficient (90%, 95% and 99%). The model ignores the possibility of extinction, a risk that may be high under individual and/or temporal (e.g., due to climate) variance in reproductive success. After 6000 generations, 13.5 - 24% % runs resulted in fixation of the adaptive variant (a variant that eradicates the vernalization requirement) (Supplementary Table 8). The time of the simulated trajectories was inverted to a backwards in time model to follow the same structure as the inferred trajectory from CLUES. Supplementary Figure 14 shows the trajectories of functional variants that arise under the three different selfing coefficients along with the inferred trajectory.

Santo Antão fits somewhat less well with the idealized model due to its more complex history. The colonization event in Santo Antão is confounded with the much earlier split from the ‘ghost’ population. Further, population structure appears to have developed in Santo Antão well before FRI 232X appeared, consistent with a more permissive landscape where late-flowering plants could survive to reproduce potentially with moderate to high success in some years. We consider a range of possible N_e from 500 to 1000 based on the estimated N_e in Santo Antão at the time when FRI 232X arose. We examine the scenario with *s* set to 0.046 based on the selection coefficient inferred from the frequency trajectory of FRI 232X in Santo Antão during the peak of selection. U_b is the same as in the Fogo case.

With these assumptions $4N_eU_b$ would range from 3.08×10^{-3} to 6.16×10^{-3}, which is again much less than 1.0, indicating limited mutational input for adaptation. And *s* of 0.046 is much greater than $1/4N_e$, which ranges from 5×10^{-4} to 2.5×10^{-4}, consistent with expectations for a SSWM model, albeit less extreme than the Fogo case.

Given that most observed large changes in flowering time in nature can be attributed to changes in *FRI* and *FLC* (85%)[109,152,156–159], it seems reasonable to focus on these genes in our analysis of mutational target size. But there are many genes across the genome that act in the flowering time pathway and through which variation could affect flowering time. If we broadened the phenotypic definition to include variants with less extreme effects on flowering

time, the architecture of the trait would be more polygenic and U_b would be larger. A few other genes are known to have fairly large effects on flowering time (ranging from 6 to 10 days) and act through vernalization (e.g., *FLM*, genes in the *MAF2-5* gene cluster, and *SVP*). We could attempt to calculate expected waiting times for any change in flowering time under progressively more polygenic architectures. While we have no estimate of *s* to which we could compare in these cases, we can assume that it would be smaller, and that drift would then play a much more important role in the probability that mutations in these genes would be established in the population. In a larger population, where new variants were less susceptible to loss by drift, the first steps of adaptation may be more likely to include variants in genes with weaker effects.

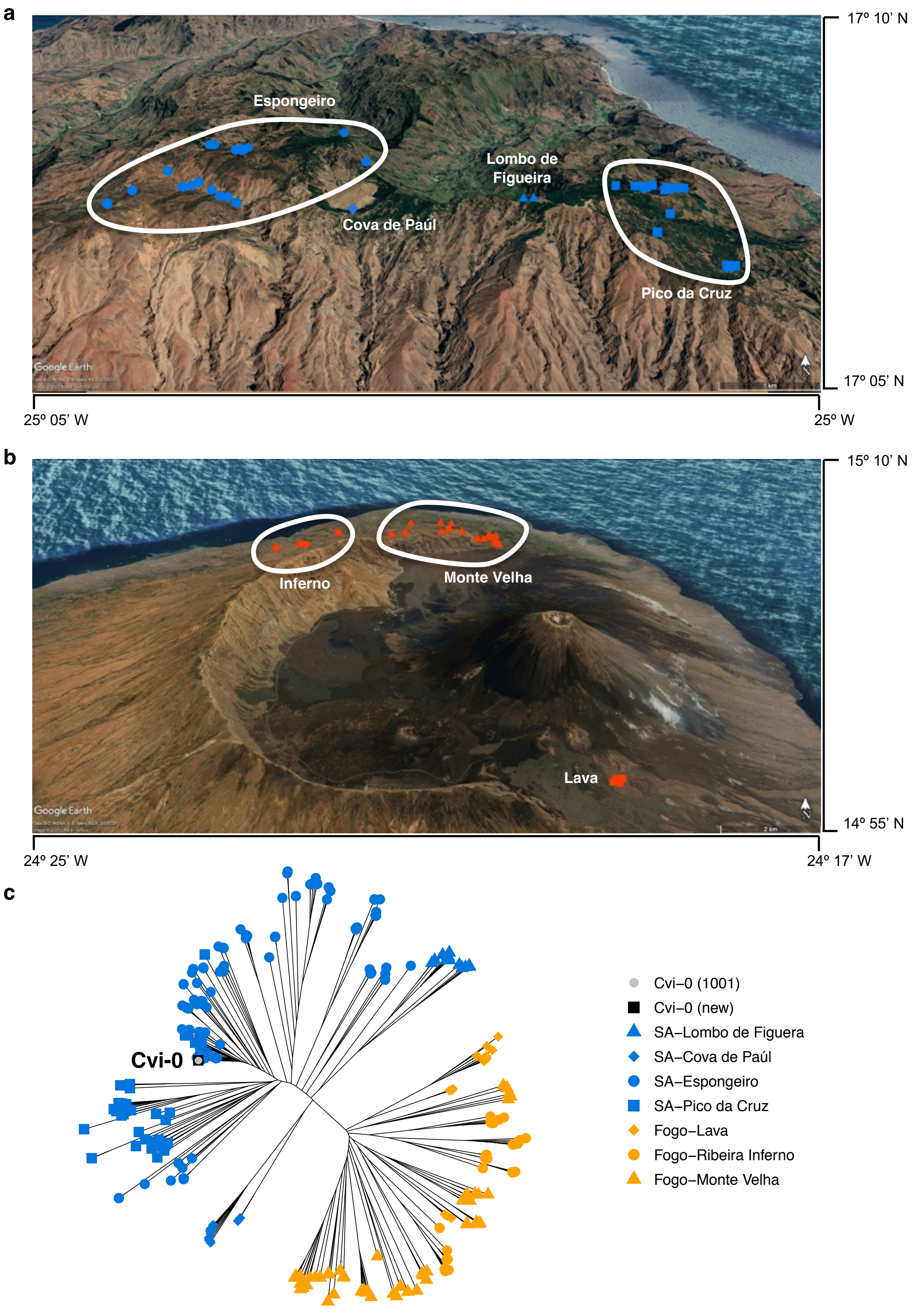
a
Espongeiro
Lombo de Figueira
Cova de Paúl
Pico da Cruz
17º 10’ N
17º 05’ N
25º 05’ W
25º W
b
Inferno
Monte Velha
Lava
15º 10’ N
14º 55’ N
24º 25’ W
24º 17’ W
c
Cvi-0
Cvi–0 (1001)
Cvi–0 (new)
SA–Lombo de Figuera
SA–Cova de Paúl
SA–Espongeiro
SA–Pico da Cruz
Fogo–Lava
Fogo–Ribeira Inferno
Fogo–Monte Velha

Supplementary Figure 1. Geographic locations and genetic clustering of CVI samples. Maps of sub-populations in **a**, Santo Antão and **b,** Fogo. Maps were created using Google Earth basemaps [160,161]. **c,** Neighbour-joining tree showing deep separation between islands and clustering of island sub-populations. Cvi-0 clusters with the Santo Antão population. Samples from Santo Antão are shown in blue, with sub-populations denoted as follows: Lombo de Figueira (triangles), Cova de Paúl (diamonds), Espongeiro (circles), and Pico da Cruz (squares). Samples from Fogo are shown in orange, with sub-populations denoted as Lava (diamonds), Ribeira Inferno (circles), and Monte Velha (triangles). Cvi-0 (1001) represents the Cvi-0 sequenced in the 1001 Genomes Project for *Arabidopsis thaliana*, while Cvi-0 (new) represents the Cvi-0 re-sequenced in this study. Source data are provided as a Source Data file.

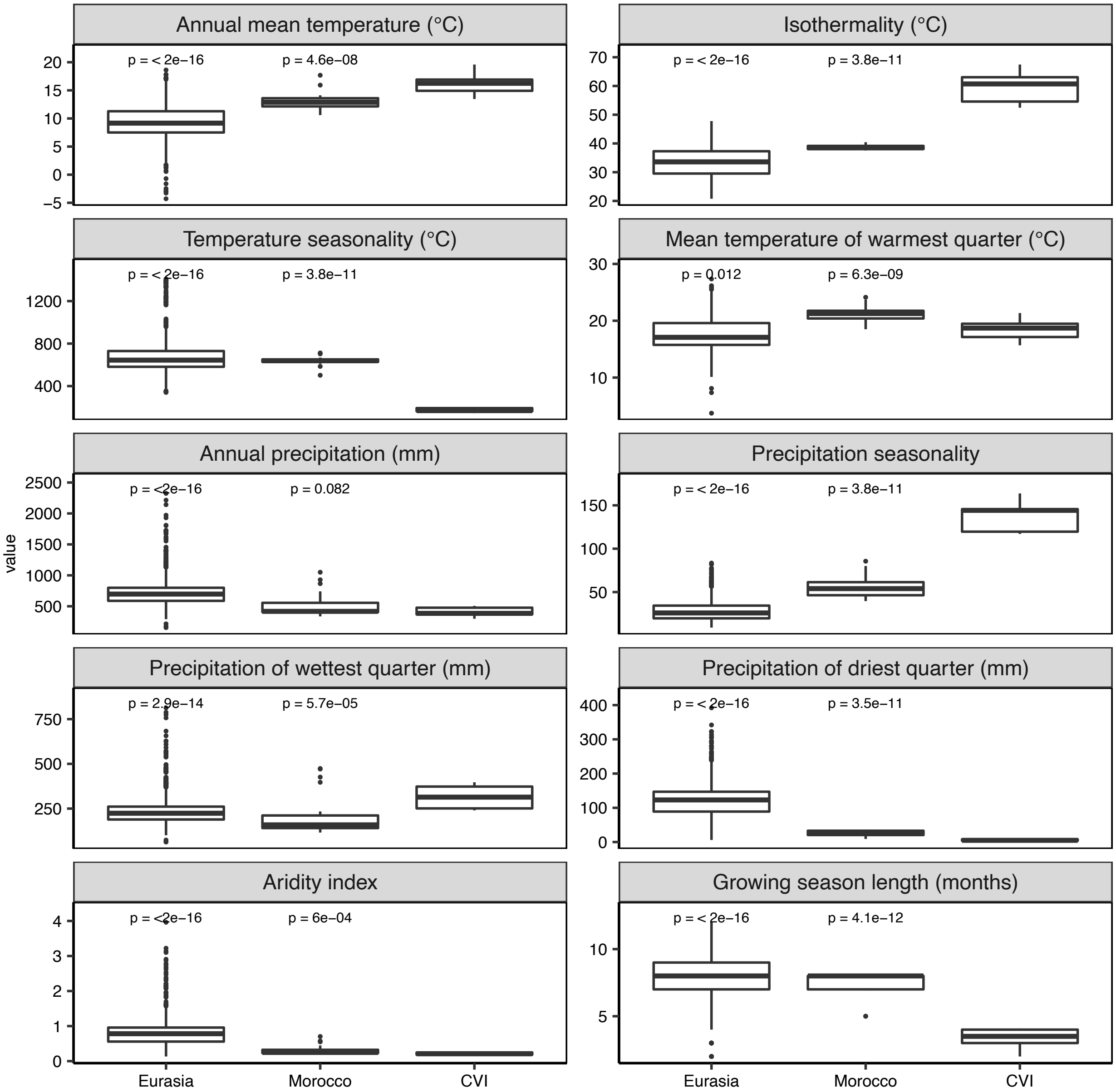

Supplementary Figure 2. Climate at collection sites in CVI relative to Moroccan and Eurasian sites. The three populations are shown on the x-axis, while the values for each climatic variable are shown on the y-axis. Each variable is presented with the respective unit. Boxplots

show median, 1st and 3rd quartiles, and whiskers represent 95% CI. *P*-values for two-sided Wilcoxon tests are shown for Morocco (n=20) and Eurasia (n=1060) relative to CVI (n=56). Annual mean temperature: Eurasia: p-value < 2x10^{-16}, Morocco: p-value = 4.6x10^{-8}; Isothermality: Eurasia: p-value < 2x10^{-16}, Morocco: p-value = 3.6x10^{-11}; Temperature seasonality: Eurasia: p-value < 2x10^{-16}, Morocco: p-value = 3.8x10^{-11}; Mean temperature of the warmest quarter: Eurasia: p-value = 0.012, Morocco: p-value = 6.3x10^{-9}; Annual precipitation: Eurasia: p-value < 2x10^{-16}, Morocco: p-value = 0.082; Precipitation seasonality: Eurasia: p-value < 2x10^{-16}, Morocco: p-value = 3.8x10^{-11}; Precipitation of the wettest quarter: Eurasia: p-value = 2.9x10^{-14}, Morocco: p-value = 5.7x10^{-5}; Precipitation of driest quarter: Eurasia: p-value < 2x10^{-16}, Morocco: p-value = 3.5x10^{-11}; Aridity index: Eurasia: p-value < 2x10^{-16}, Morocco: p-value = 6x10^{-4}; Growing season length: Eurasia: p-value < 2x10^{-16}, Morocco: p-value = 4.1x10^{-12}. Source data are provided as a Source Data file.

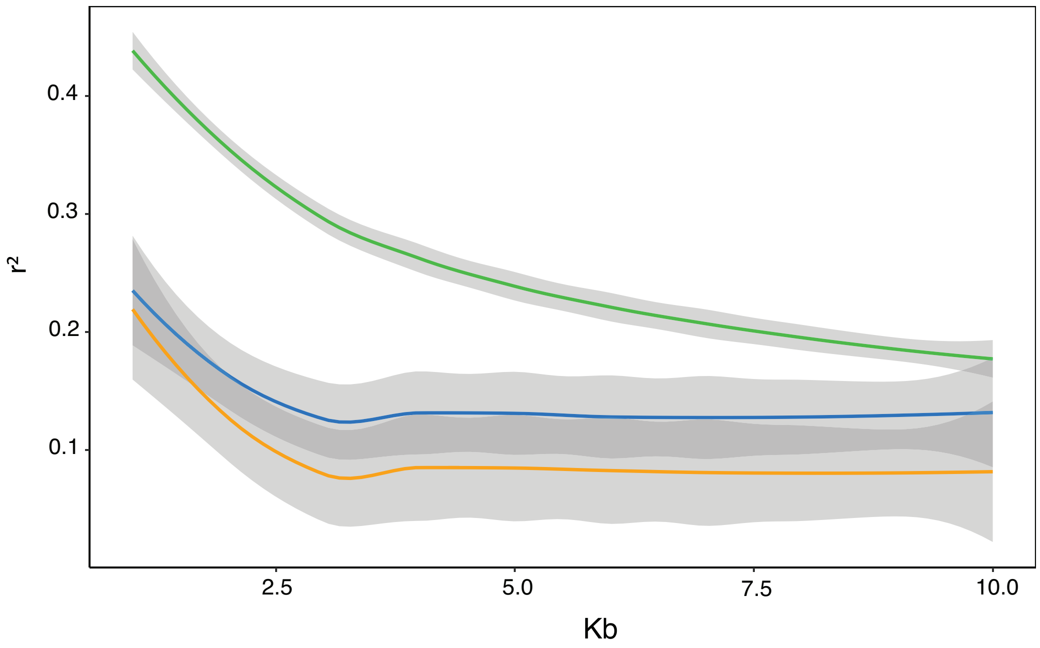

Supplementary Figure 3. Linkage disequilibrium (LD) decay in all three populations. X-axis shows distance between SNPs in kbp, and the y-axis the corresponding r^2 value. Decay of LD is shown for Santo Antao (blue), Fogo (orange) and Morocco (green). Lines were smoothed with locally weighted scatterplot smoothing (LOESS) and the shaded grey area represents the 95% confidence interval.

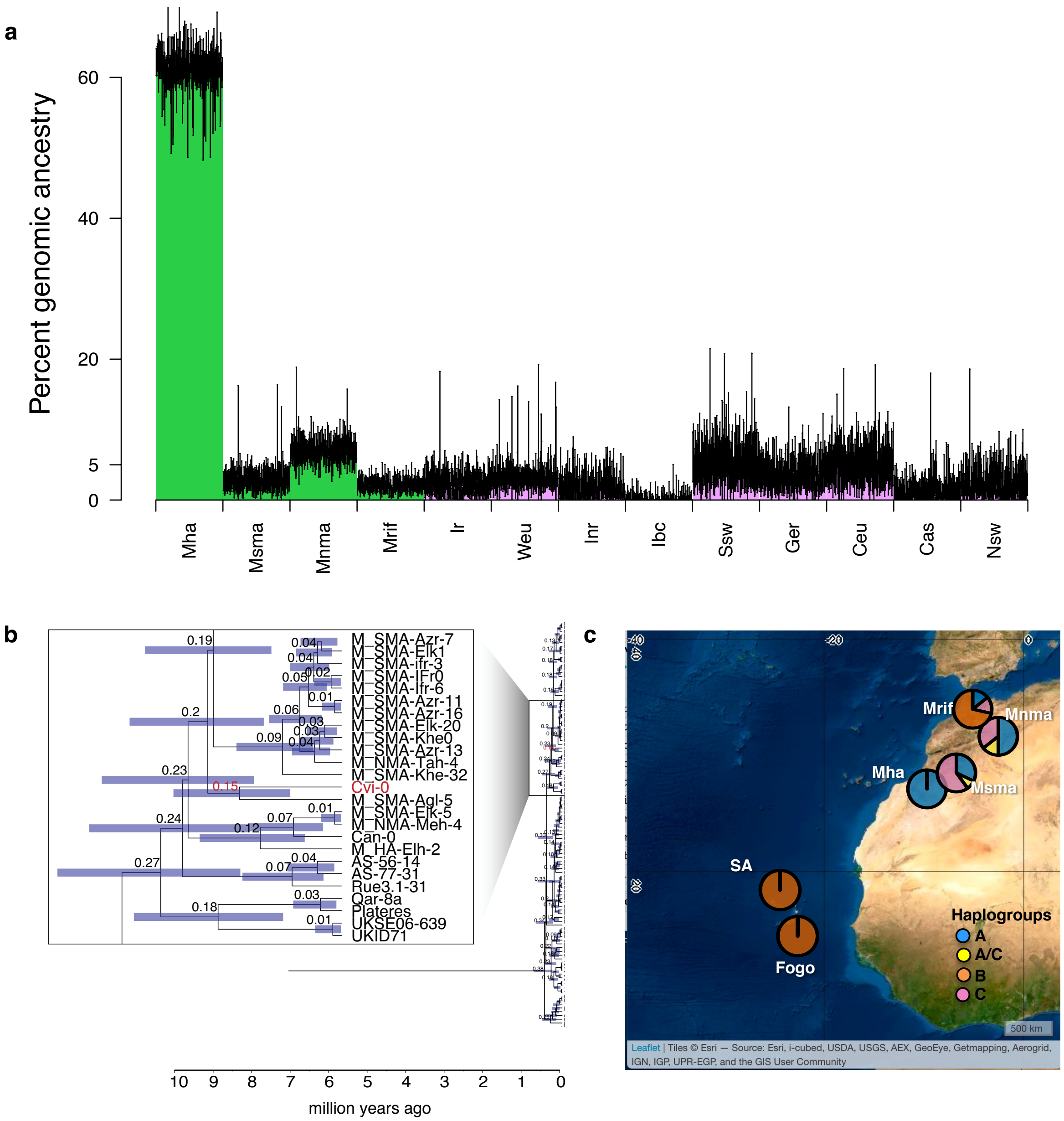

Supplementary Figure 4. Local ancestry of CVI genomes. a, The percentage of CVI genomes for which the closest relative is found in each continental population, inferred with Chromopainter. Averages across runs are shown as coloured bars, 95% confidence interval (CI) as vertical black lines, one for each of 148 CVI genomes. Genome-wide, the Moroccan

population in the High Atlas Mountain is the closest relative for 61% of CVI genomes. **b,** Region of the chloroplast phylogeny with Cvi-0 and worldwide accessions (Full phylogeny in Supplementary Fig. 5). Cvi-0 clusters with the Moroccan population from South Middle Atlas with an estimate of 150 ky divergence time from the closest Moroccan individual. Numbers at the nodes are divergence time in million years. Purple shades represent the 95% CI. **c,** Geographic distribution of S-locus haplogroups in Morocco and CVI. All individuals from the islands carry haplogroup B, which is only found in the Rif population in Morocco. Different colours in the pie charts represent different haplogroups: A in blue, A/C in yellow, B in orange, and C in pink. Abbreviations: Mha, Moroccan High Atlas; Msma, Moroccan South Middle Atlas; Mnma, Moroccan North Middle Atlas; Mrif, Moroccan Rif; Ir, Iberian relicts; Weu, Western Europe; Inr, Iberian non-relicts; Ibc, Italy, Balkans and Caucasus; Ssw, South Sweden; Ger, Germany; Ceu, Central Europe; Cas, Central Asia; Nsw, North Sweden; SA, Santo Antão. Basemap was retrieved from the World Imagery map[162] using leaflet[163]. Source data are provided as a Source Data file.

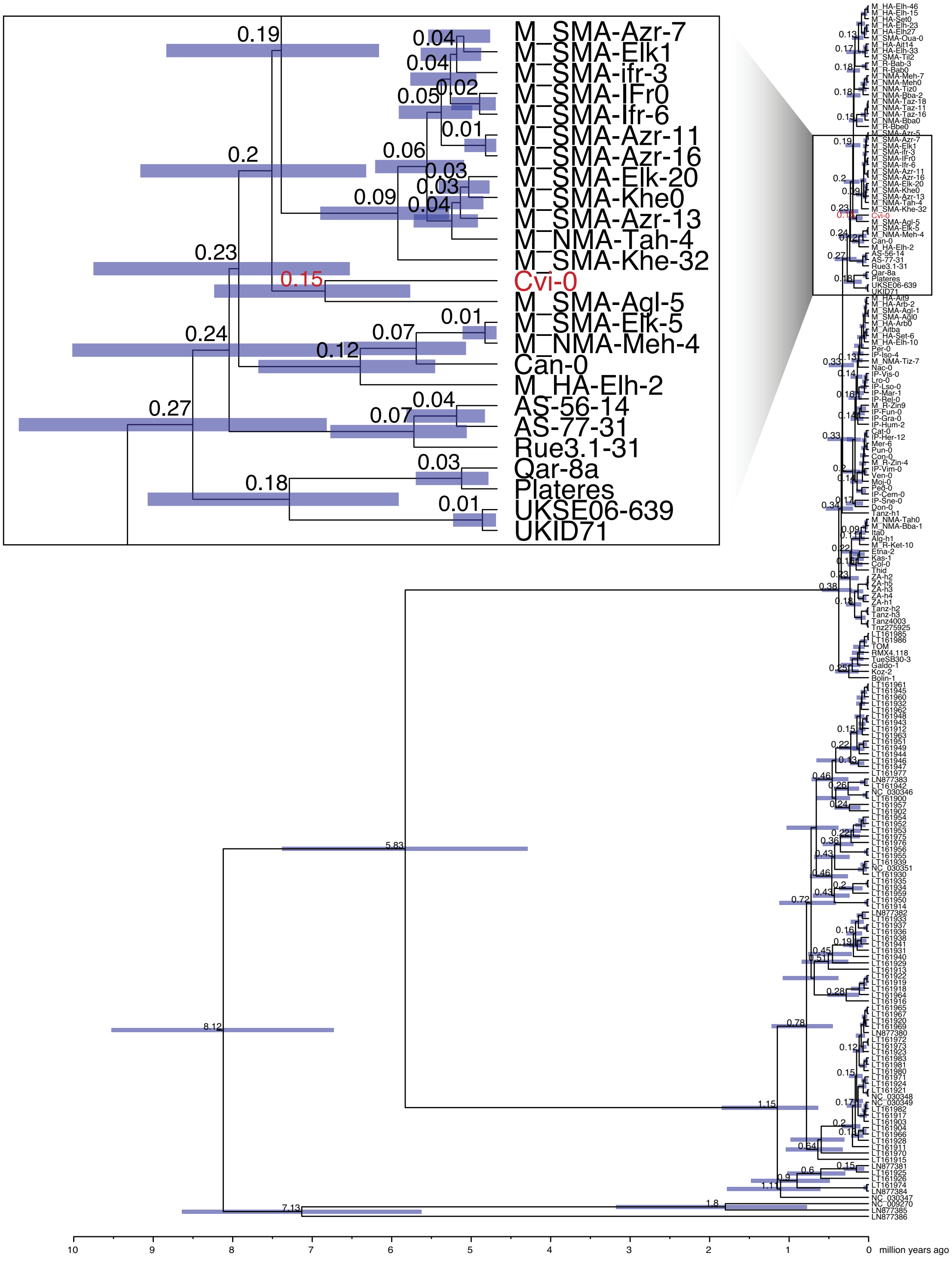
M_SMA-Azr-7
M_SMA-Elk1
M_SMA-ifr-3
M_SMA-IFr0
M_SMA-Ifr-6
M_SMA-Azr-11
M_SMA-Azr-16
M_SMA-Elk-20
M_SMA-Khe0
M_SMA-Azr-13
M_NMA-Tah-4
M_SMA-Khe-32
Cvi-0
M_SMA-Agl-5
M_SMA-Elk-5
M_NMA-Meh-4
Can-0
M_HA-Elh-2
AS-56-14
AS-77-31
Rue3.1-31
Qar-8a
Plateres
UKSE06-639
UKID71
0.19
0.2
0.23
0.15
0.24
0.27
0.18
0.09
0.12
0.07
5.83
8.12
7.13
1.15
1.8
10
9
8
7
6
5
4
3
2
1
0
million years ago

Supplementary Figure 5. Time-calibrated chloroplast phylogeny showing the location of Cvi-0 relative to representatives of *A. thaliana*, and other *Arabidopsis* species, *Capsella grandiflora*, *Capsella bursa-pastoris* as well as *Camelina sativa* for calibration. Inset shows the location of Cvi-0.

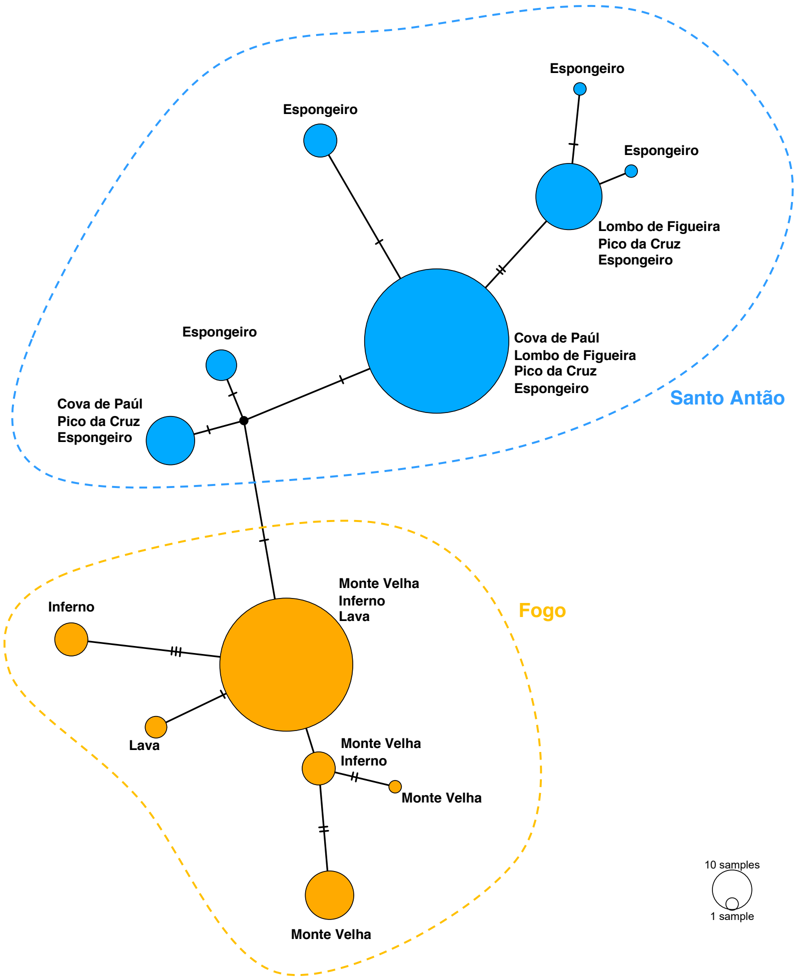

Supplementary Figure 6. Chloroplast network for all CVI accessions. The size of the nodes is proportional to the number of samples, with the corresponding cluster name. Blue represents haplotypes in Santo Antão, orange in Fogo.

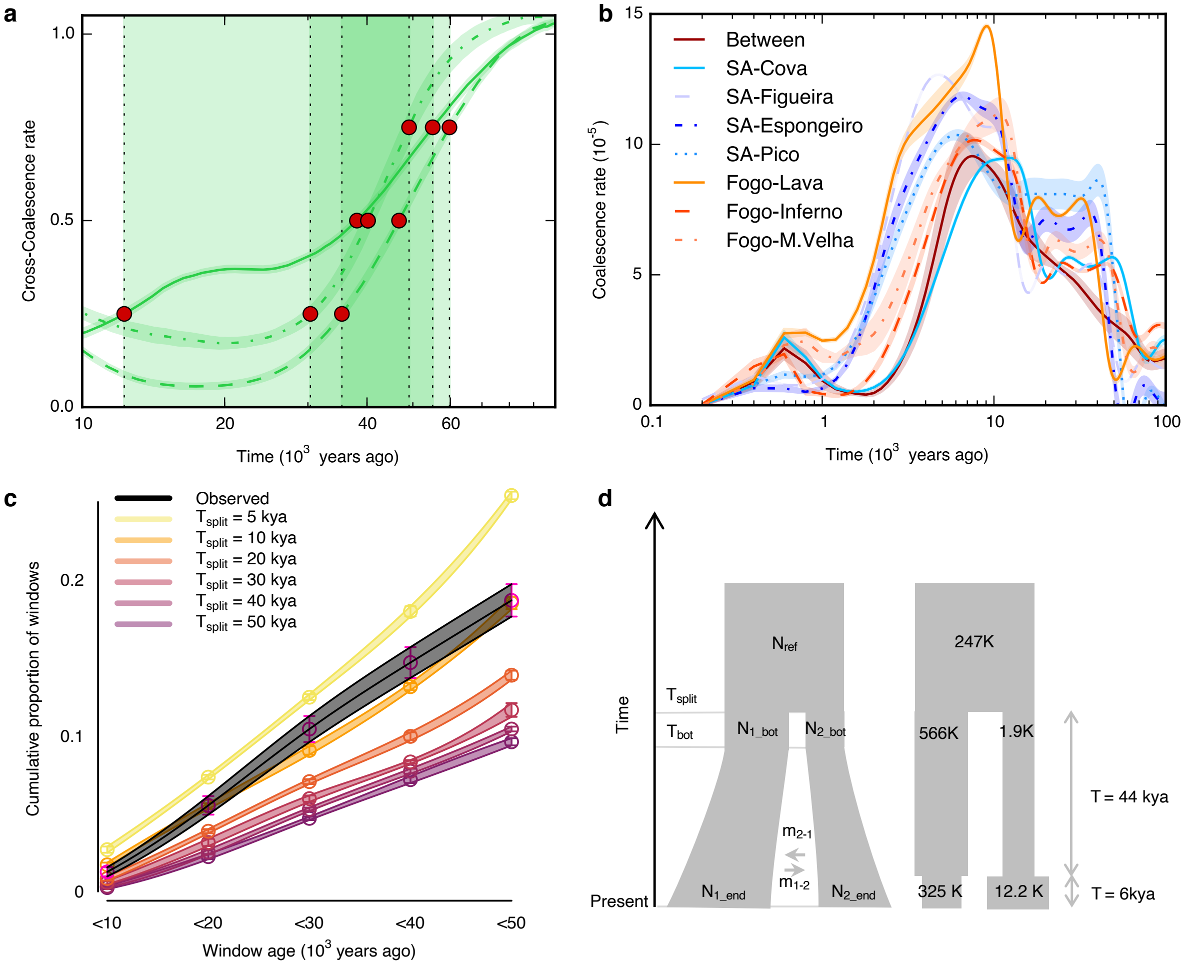

Supplementary Figure 7. Modelling of Morocco-CVI split. a, Split time estimated with MSMC-CCR for the Moroccan populations and CVI. Solid line represents the High Atlas population, dashed line the North Middle Atlas and dash-dotted line the South Middle Atlas populations. 0.25 – 0.75 cross coalescence rate quantiles are shown as shaded areas, with point estimates at 0.25, 0.5 and 0.75 highlighted by red dots. **b,** Coalescence rate as a function of time within Santo Antão and Fogo clusters, and between islands (‘Between’), estimated with MSMC2 in 8-haplotypes mode. Lines represent means across octets, shaded areas the 95% CI. **c,**

Cumulative proportion of genomic windows with different inferred ages based on the density of mutations. Simulations were run with a split at different times (5-50 kya) between a Morocco-like and CVI-like population. Points represent observed data and averages across simulations. Whiskers represent 95% CI based on the SE estimated with ordinary non-parametric bootstrap for observed data and on the SE across simulations. Points were interpolated with cubic splines. Simulations with a split at 10 kya most closely match the observed data, supporting a colonization of CVI from a 'ghost' population more recent than 10 kya. **d,** Overview of models run in dadi (left) and best model (right). N_{ref}, size of the ancestral population; N_{1_bot}, N_{2_bot}, size of population one and two at the split, respectively; only in the bottleneck models, N_{1_bot} and N_{2_bot} remain constant for the duration of the bottleneck, T_{bot}; N_{1_end}, N_{2_end}, population sizes at present; T_{split}, split time; m_{12}, m_{21}, migration rates. Estimates of the split time with MSMC-CCR reveal a complex scenario, likely due to the absence of a direct outgroup to CVI in our sample. In these analyses, there is evidence of a split at 40-50 kya, followed by secondary contact or low-level migration between 10 and 20 kya. This initial split and secondary contact likely happened on the continents between sampled Moroccan populations and an unsampled 'ghost' population (the true closest continental outgroup to CVI). Source data are provided as a Source Data file.

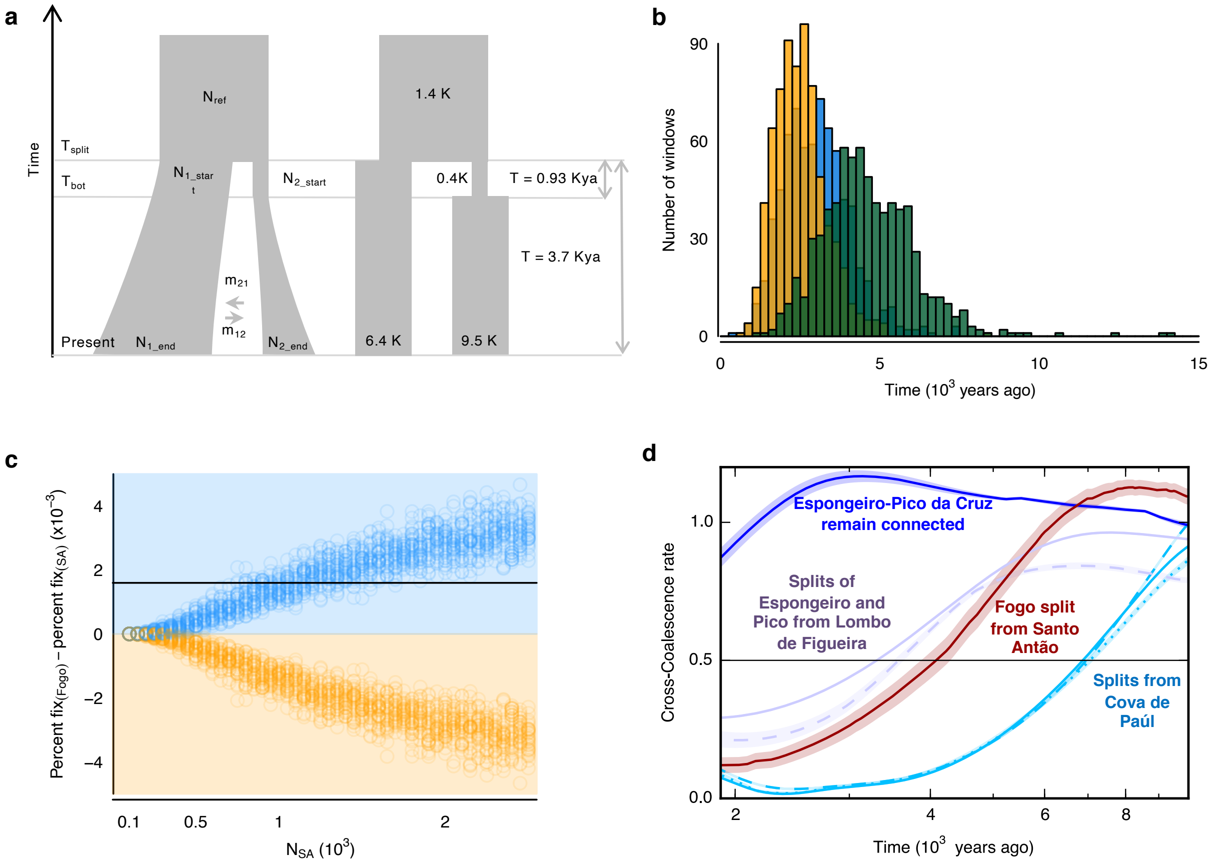

Supplementary Figure 8. Modelling the dynamics within CVI. a, Overview of models run in dadi (left) and best model (right). N_{ref}, size of the ancestral population; N_{1_start}, N_{2_start} , size of population one and two at the split, respectively; only in the bottleneck model, N_{2_start} remains constant for the duration of the bottleneck, T_{bot}; N_{1_end}, N_{2_end}, population sizes at present; T_{split}, split time; m_{12}, m_{21}, migration rates. **b,** Coalescence time within CVI across genomic windows (size=0.1Mbp) provides a lower bound for colonization timing at approximately 7 kya (95th percentile of coalescence times between islands). Comparisons within island are in blue (Santo Antão) and orange (Fogo), and comparisons between islands in green. **c,** Forward simulations

fitted to observed data support a scenario in which Santo Antão was colonized prior to Fogo. The percentage of fixed differences in Fogo compared to Santo Antão (SA) minus the percentage of fixed differences in Santo Antão compared to Fogo (y-axis) varies as a function of which island was colonized first (positive if Santo Antão, blue shade; negative if Fogo, orange shade) and of the population size at the split (x-axis). Circles represent the results from simulations (n=1516): blue if Santo Antão was colonized first, orange if Fogo was colonized first. Simulations in which Santo Antão was colonized first with a population size of approximately 1K at the time of the split, and in which Fogo was colonized later from Santo Antão, fit best the observed data (horizontal black line). **d,** Cross-coalescence rates (CCR) among populations in Santo Antão as inferred by MSMC-CCR. The estimated between-population split time corresponds to CCR=0.5 (horizontal line). Dark red shows CCR between Santo Antão and Fogo for reference. Lines represent means across octets, shaded areas the 95% CI. Source data are provided as a Source Data file.

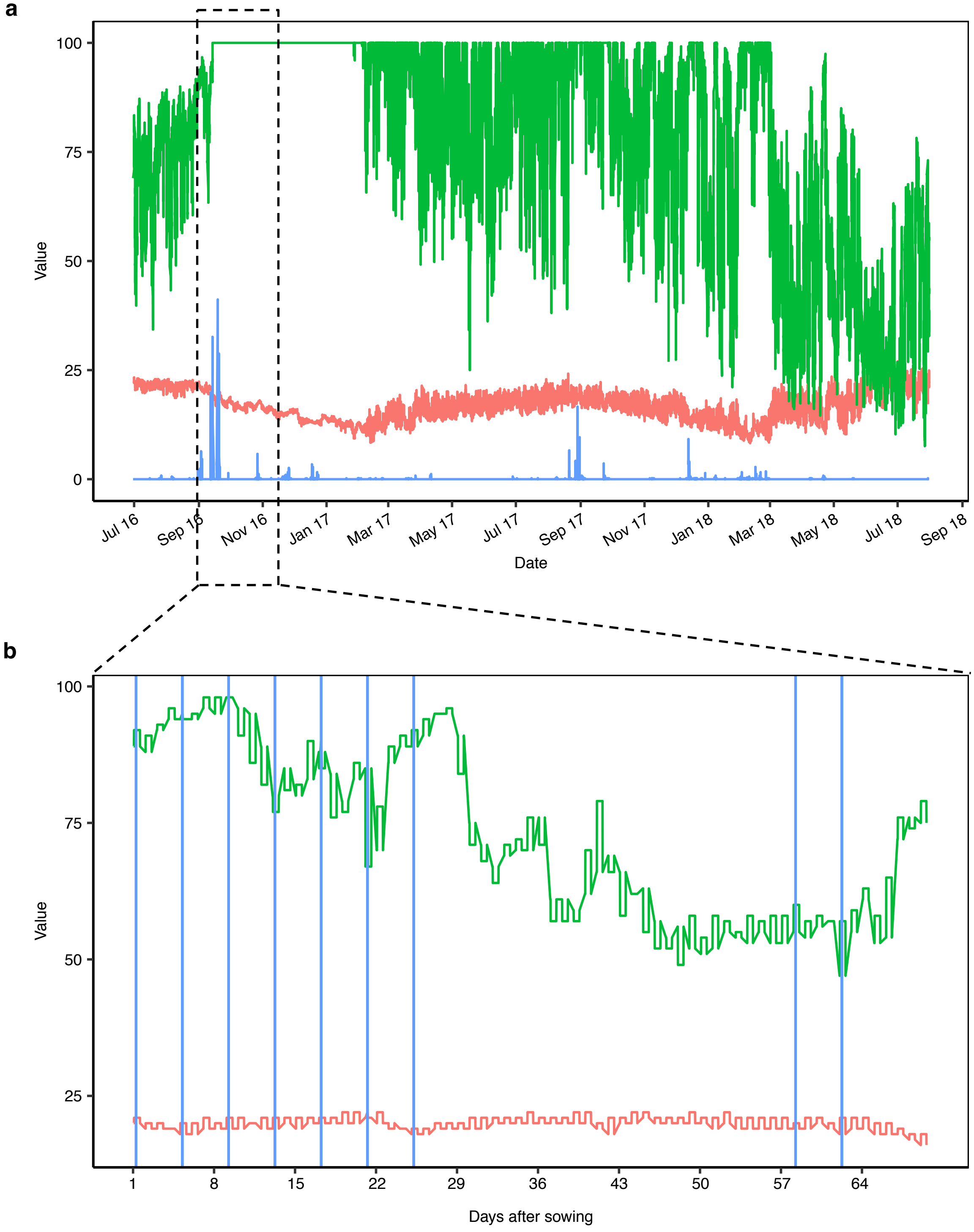
a
b
Value
100
75
50
25
0
Date
Jul 16
Sep 16
Nov 16
Jan 17
Mar 17
May 17
Jul 17
Sep 17
Nov 17
Jan 18
Mar 18
May 18
Jul 18
Sep 18
Days after sowing
1
8
15
22
29
36
43
50
57
64

1112

Supplementary Figure 9. Field climate data and simulated conditions. a, Field site measurements for precipitation (blue), humidity (green) and temperature (red) using loggers in the Espongeiro field site over two years (July 2016 to July 2018). Y-axis shows values in mm for precipitation, in percentage for humidity and in degrees Celsius for temperature. The dashed line highlights the period of time simulated in panel b. **b,** Chamber conditions during the experiment for humidity (green) and temperature (red). Watering is shown as blue vertical lines. Y-axis shows values in percentage for humidity and in degrees Celsius for temperature, and x-axis shows days after sowing (day 1: 1st Sept 2016). Source data are provided as a Source Data file.

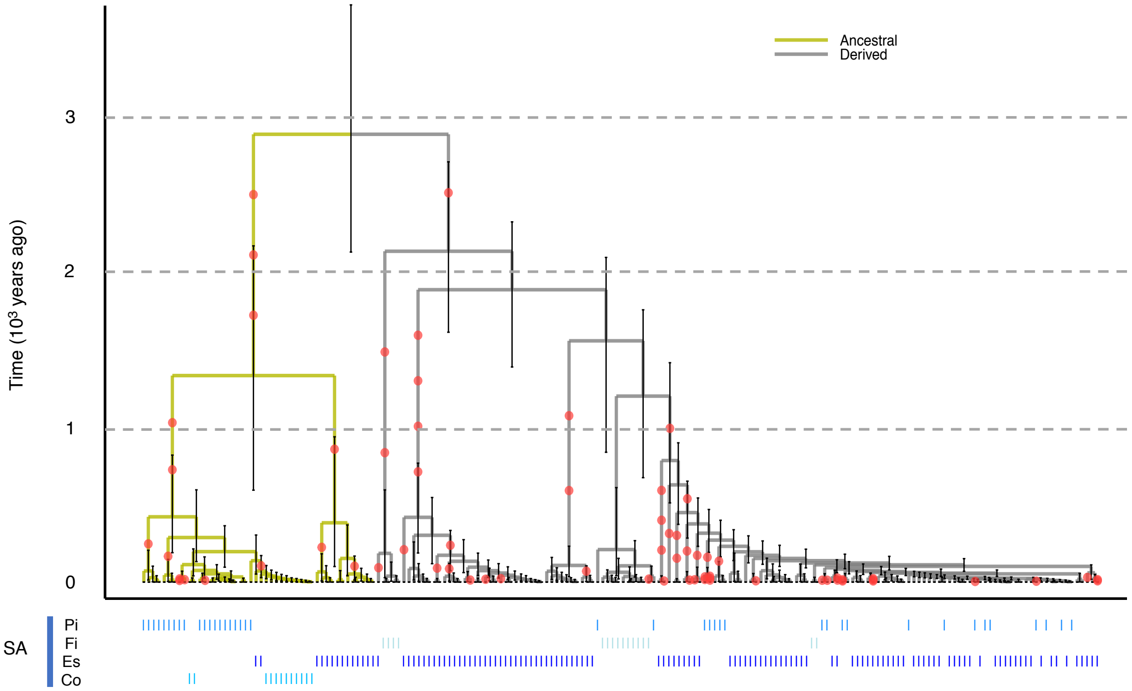

Supplementary Figure 10. Evolutionary history of FRI K232X. Marginal genealogical tree estimated in RELATE for *FRI* (Chr4:269719). Individuals are shown across the x-axis with their ancestral and derived carriers coloured in mustard and grey, respectively. The estimated time to coalescence is shown on the y-axis. Error bars indicate the 0.025 and 0.975 quantiles of the posterior density of coalescence times and are shown by the vertical black lines on the tree. Red dots represent mapped SNPs on their corresponding branches. Abbreviations: SA, Santo Antão; Pi, Pico da Cruz; Fi, Lombo de Figueira; Es, Espongeiro; and Co, Cova de Paúl. Source data are provided as a Source Data file.

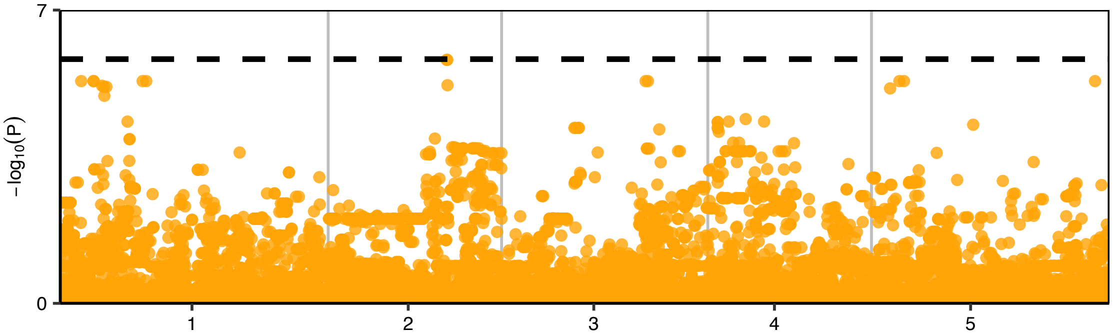

Supplementary Figure 11. GWAS in Fogo. Manhattan plot showing results of GWAS for bolting time in the Fogo population (n=129). Dashed line represents the Bonferroni significance threshold. Source data are provided as a Source Data file.

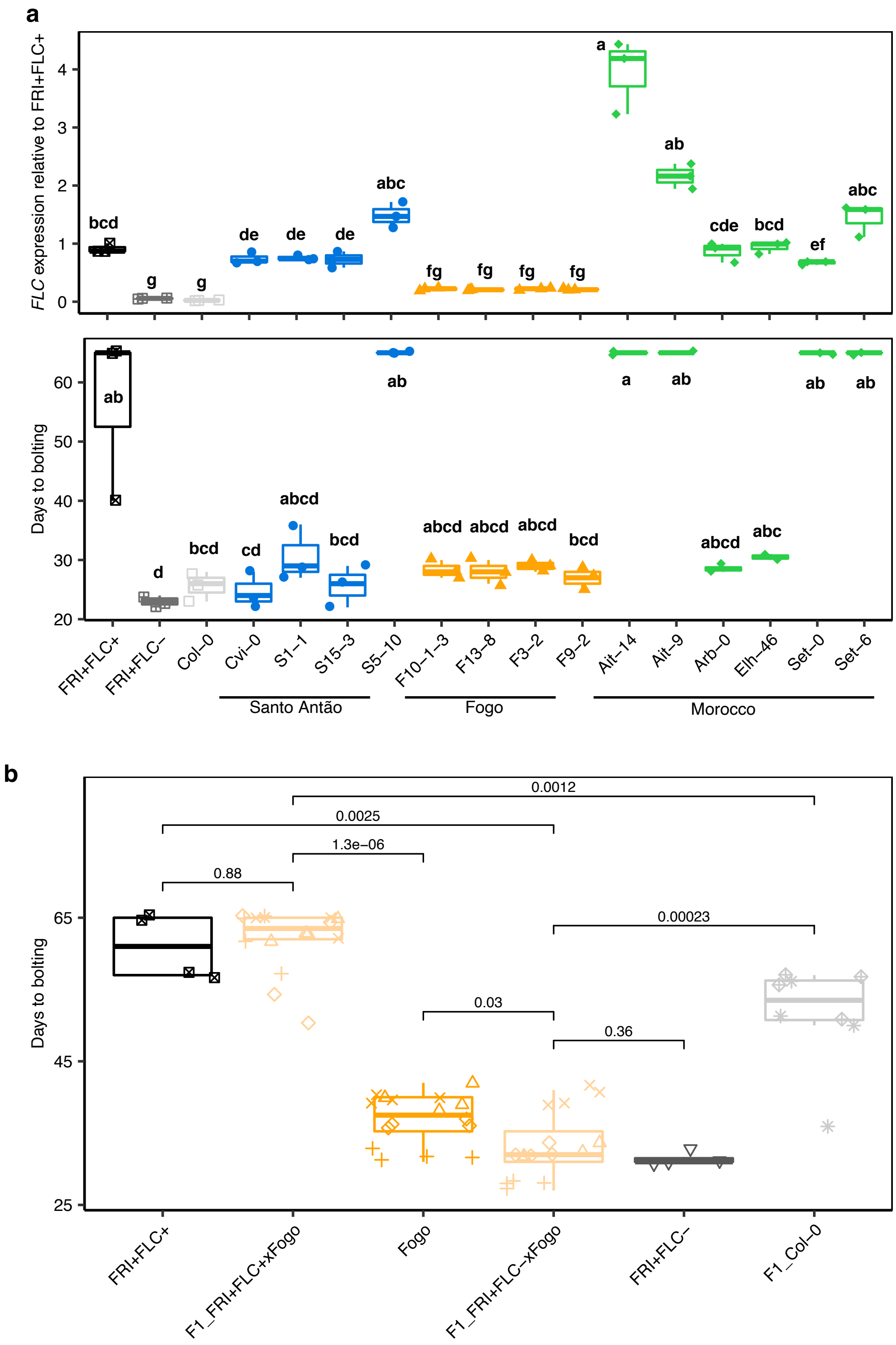
a
FLC expression relative to FRI+FLC+
Days to bolting
bcd
g
g
de
de
de
abc
fg
fg
fg
fg
a
ab
cde
bcd
ef
abc
ab
ab
a
ab
ab
ab
d
bcd
cd
abcd
bcd
abcd
abcd
abcd
bcd
abcd
abc
FRI+FLC+
FRI+FLC−
Col−0
Cvi−0
S1−1
S15−3
S5−10
F10−1−3
F13−8
F3−2
F9−2
Ait−14
Ait−9
Arb−0
Elh−46
Set−0
Set−6
Santo Antão
Fogo
Morocco
b
Days to bolting
0.0012
0.0025
1.3e−06
0.88
0.00023
0.03
0.36
FRI+FLC+
F1_FRI+FLC+xFogo
Fogo
F1_FRI+FLC−xFogo
FRI+FLC−
F1_Col−0

1138

Supplementary Figure 12. Functional characterization of *FLC* nonsense mutation. a, *FLC* effect on natural accessions from CVI and Morocco, and in mutant lines in the Col-0 background. FRI K232X segregates among the Santo Antão individuals shown. Cvi-0, S1-1 and S15-3 carry the FRI 232X allele, while S5-10 carries the ancestral FRI K232 allele. All Fogo individuals carry the derived FLC 3X allele. Top: *FLC* mRNA expression levels, with y-axis showing the expression levels relative to functional FRI FLC ($FRI^{+}FLC^{+}$). Bottom: Days to bolting (shown on the y-axis). In both panels, dots represent replicates per genotype (n=3), and letters above boxplot represent statistical groups from Kruskal Wallis tests. The central line in the boxplots represents the median, box limits are first and third quartiles and whiskers the 95% CI. **b,** Bolting time differences in the complementation test. Each dot represents one replicate per genotype (n=4), and different symbols represent different accessions, with symbols matching between Fogo and F1 for the parental lines. The central line in the boxplots represents the median, box limits are first and third quartiles and whiskers the 95% CI. *P*-values are shown for two-sided Wilcoxon test. Throughout the figure, $FRI^{+}FLC^{+}$ represents the accession Col-0 *FRI*-Sf2, with both functional FRI and FLC; $FRI^{+}FLC^{-}$ represents the accession Col-0 *FRI*-Sf2 *flc-3*, carrying a functional FRI and a non-functional FLC; F1_$FRI^{+}FLC^{+}$xFogo represents F1 individuals from crosses between $FRI^{+}FLC^{+}$ and Fogo accessions; F1_$FRI^{+}FLC^{-}$xFogo represents F1 individuals from crosses between $FRI^{+}FLC^{-}$ and Fogo accessions; F1_Col-0 represents F1 individuals from crosses between $FRI^{+}FLC^{+}$ and $FRI^{+}FLC^{-}$. Comparisons *p*-values: $FRI^{+}FLC^{+}$ *vs.* F1_$FRI^{+}FLC^{+}$xFogo = 0.88, F1_$FRI^{+}FLC^{+}$xFogo vs. Fogo = 1.3×10^{-6}, $FRI^{+}FLC^{+}$ vs. F1_$FRI^{+}FLC^{-}$xFogo = 0.0025, F1_$FRI^{+}FLC^{+}$xFogo vs. F1_Col-0 = 0.0012, Fogo vs,

F1_*FRI*$^{+}$*FLC*$^{-}$xFogo = 0.03, F1_*FRI*$^{+}$*FLC*$^{-}$xFogo vs. *FRI*$^{+}$*FLC*$^{-}$ = 0.36, F1_*FRI*$^{+}$*FLC*$^{-}$xFogo vs. F1_Col-0 = 0.00023. For both panels, boxplots show median (centre), 1st and 3rd quartiles (lower and upper bound, respectively). Whiskers represent 95% CI. Source data are provided as a Source Data file.

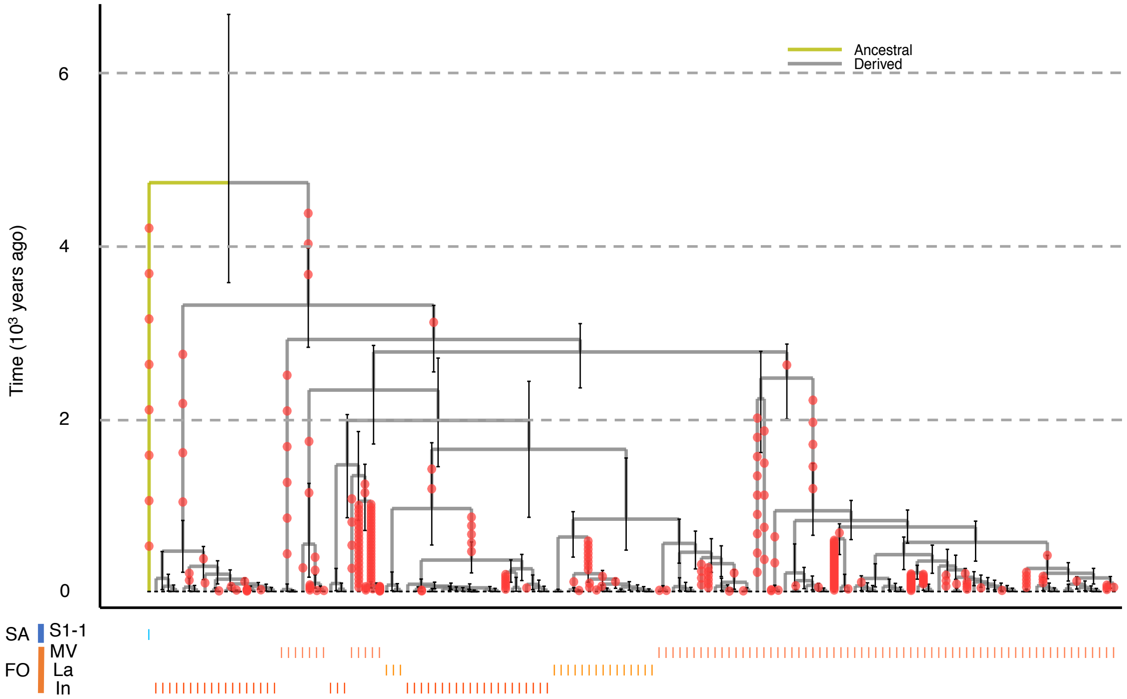

Supplementary Figure 13. Marginal genealogical tree estimated in RELATE for FLC 3X (Chr5: 3179333). Individuals are shown across the x-axis with the ancestral and derived carriers coloured in mustard and grey, respectively. The estimated coalescence times are shown on the y-axis. Error bars indicate the 0.025 and 0.975 quantiles of the posterior density of coalescence times and are shown by the vertical black lines on the tree. Red dots represent mapped SNPs on their corresponding branches. Abbreviations: FO, Fogo; MV, Monte Velha; La, Lava; In, Inferno; SA, Santo Antão; and S1-1 accession from Lombo de Figueira subpopulation in Santo Antão (used as the outgroup). Source data are provided as a Source Data file.

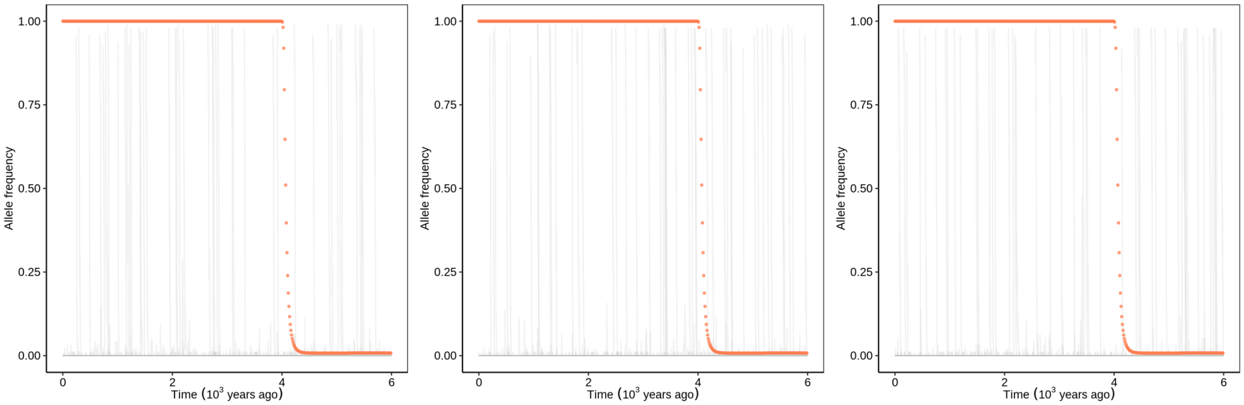

Supplementary Figure 14. Allele frequency trajectories of variants arising in a simulated Fogo colonizing population. Simulated allele frequency trajectories of variants arising in Fogo (starting population size=48) with selection coefficients of 9.2% (the estimate from CLUES with variable N_e). Each grey line represents a simulated trajectory, and the orange line represents the trajectory inferred from CLUES for FLC R3X, across selfing rates (left to right: 90%, 95%, 99%).

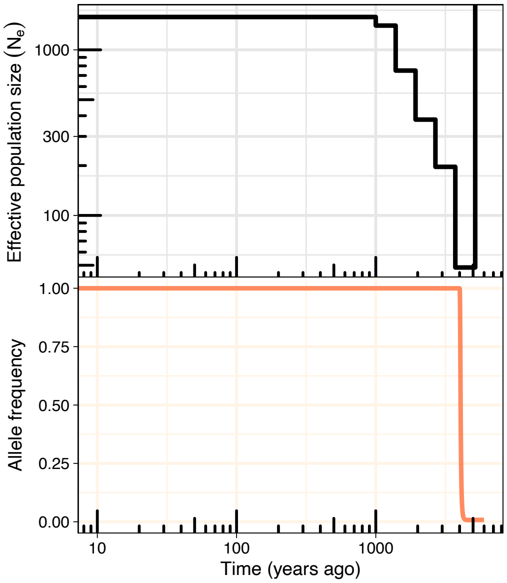

Supplementary Figure 15. FLC 3X appeared in Fogo soon after colonization, and rose in frequency rapidly, consistent with evolutionary rescue. Top: reconstructed change in effective population size over time in Fogo. Bottom: inferred allele frequency trajectory of FLC 3X. Source data are provided as a Source Data file.

Supplementary Table 1. Nucleotide diversity (θ) in Cape Verde, North Africa and Eurasia.

	**θ_π**[1]	**θ_w**[2]
CVI	$6.53x10^{-5}$	$1.48x10^{-4}$
Santo Antão	$5.60x10^{-5}$	$7.59x10^{-5}$
Fogo	$4.51x10^{-5}$	$8.93x10^{-5}$
Morocco	$5.38x10^{-3}$	$5.56x10^{-3}$
High Atlas	$3.21x10^{-3}$	$2.72x10^{-3}$
North Middle Atlas	$2.74x10^{-3}$	$2.27x10^{-3}$
South Middle Atlas	$3.53x10^{-3}$	$3.41x10^{-3}$
Rif	$4.69x10^{-3}$	$4.00x10^{-3}$
Eurasia	$4.21x10^{-3}$	$6.42x10^{-3}$

[1] $\theta\pi$: Tajima's estimator of θ

[2] θw: Watterson's estimator of θ.

Supplementary Table 2. Modelling of the split between Morocco and CVI with dadi.

Model	MaxLik[1]	AIC[2]	Theta[3]	Nref[4]	N1_start[5]	N2_start[6]	N1_end[7]	N2_end[8]	Tsplit[9]	m12[10]	m21[11]	Tbot[12]
simpleSplit	-461	928	1878	239122	447059	2876	x	x	69802	x	x	x
Exp	-376	758	2039	259618	258831	787	555769	11967	55731	x	x	x
im	-1051	2112	1727	219908	4700	215208	447976	1468	1397384	7,96E-07	5,54E-06	x
bottleneck	-396	800	1849	235505	526073	253	526073	6652	50736	x	x	17041
bottleneck-2sided	-348	708	1943	247442	565501	1907	324697	12233	49668	x	x	43517

[1] MaxLik: maximum log-likelihood obtained across dadi runs
[2] AIC: Akaike Information Criterion
[3] Theta: population mutation parameter inferred in dadi
[4] Nref: effective size of the ancestral population
[5] N1_start: effective size of population one at the split, respectively. In the bottleneck models, these sizes remain constant for the duration of the bottleneck
[6] N2_start: effective size of population two at the split, respectively. In the bottleneck models, these sizes remain constant for the duration of the bottleneck
[7] N1_end: effective population size of population 1 at present
[8] N2_end: effective population size of population 2 at present
[9] Tsplit: split time
[10] m12: migration rate from population 1 to 2
[11] m21: migration rate from population 2 to 1
[12] Tbot: duration of the bottleneck

Supplementary Table 3. Modelling of the split between Santo Antão and Fogo with dadi.

Model	MaxLik[1]	AIC[2]	Theta[3]	Nref[4]	N1_start[5]	N2_start[6]	N1_end[7]	N2_end[8]	Tsplit[9]	m12[10]	m21[11]	Tbot[12]
simpleSplit	-389	784	53	776	5487	7750	x	x	4754	x	x	x
Exp	-313	635	142	2067	1579	488	9268	17166	3715	x	x	x
im	-304	620	127	1841	1427	413	9186	17069	3995	4,28E-06	6,03E-07	x
bottleneck	-282	574	99	1435	6434	397	6434	9511	3685	x	x	931

[1] MaxLik: maximum log-likelihood obtained across dadi runs
[2] AIC: Akaike Information Criterion
[3] Theta: population mutation parameter inferred in dadi
[4] Nref: effective size of the ancestral population
[5] N1_start: effective size of population one at the split, respectively. In the bottleneck models, these sizes remain constant for the duration of the bottleneck
[6] N2_start: effective size of population two at the split, respectively. In the bottleneck models, these sizes remain constant for the duration of the bottleneck
[7] N1_end: effective population size of population 1 at present
[8] N2_end: effective population size of population 2 at present
[9] Tsplit: split time
[10] m12: migration rate from population 1 to 2
[11] m21: migration rate from population 2 to 1
[12] Tbot: duration of the bottleneck

Supplementary Table 4. Niche modeling with MaxEnt for the Moroccan region.

Variable	Variable Description	Percent contribution	Permutation importance
gs	Length of the growing season in months	38,7	28,5
bio3	Isothermality (BIO2/BIO7) (×100)	20,2	43
bio6	Min Temperature of Coldest Month	18,4	11,1
bio5	Max Temperature of Warmest Month	14,5	14,7
bio7	Temperature Annual Range (BIO5-BIO6)	8	2,2
bio15	Precipitation Seasonality (Coefficient of Variation)	0,1	0,5
bio8	Mean Temperature of Wettest Quarter	0,1	0

Supplementary Table 5. Model statistics for stepwise regression analysis. The best model (nvmax = 2) identifies FRI and GI as the main contributors to fitness.

nvmax[1]	RMSE[2]	Rsquared[3]	MAE[4]	RMSE SD[5]	Rsquared SD	MAE SD
1	486,6842	0,07352437	334,8117	62,62452	0,03778863	30,72803
2	446,8749	0,2203883	311,2951	61,00429	0,05345926	29,37821
3	450,3681	0,20845137	311,7726	60,74491	0,06327362	30,66107
4	452,386	0,20253587	309,7203	60,97669	0,05890219	32,23143
5	456,163	0,1890078	316,4986	61,53983	0,06234999	33,2347
6	458,9121	0,1796467	319,3417	61,4938	0,06423225	34,29386
7	447,3371	0,22692613	305,0492	58,34149	0,0512004	31,43282

[1] nvmax: number of variables in the model
[2] RMSE: root mean square error
[3] Rsquared: squared value for R (correlation between the observed and predicted values)
[4] MAE: mean absolute error
[5] SD, standard deviation

Supplementary Table 6. Inferred selection coefficients over time for FRI 232X.

epoch	*s*
0 - 2000	0,00112
2000 - 4000	0,04558

Supplementary Table 7. Inferred selection coefficients for FLC 3X.

epoch	*S*[1]
0 - 2000	-0,99679
2000 - 4000	-0,57297
4000 - 6000	0,09273

[1] The timeframe of fixation corresponds with the epoch 4-6 kya. After fixation of the allele negative selection coefficients are generated.

Supplementary Table 8. Simulated trajectories. Each row corresponds to the summary of the simulations (n=200).

selection coefficient (*s*)	Selfing rate	% fixed[1]	Mean gen. to fixation[2]	% not fixed[3]	Mean gen. to loss[4]	% no mutations[5]	Mean gen. to 1st segregating potentially adaptive variant[6]	Mean gen. to adapt[7]
0,0927	0,9	13,5	61,37	69,5	3,48	17	2061,63	2750,7
0,0927	0,95	16,00	64,87	67,5	3,02	16,5	1980,04	3210,21
0,0927	0,99	16,00	54,71	67,5	3,19	16,5	2002,59	3000,46
0,23	0,9	21,5	34,25	58,5	2,39	20	2096,52	3224,41
0,23	0,95	23,5	33,12	61,5	2,22	15	2201,36	2714,68
0,23	0,99	24,00	33,25	52,00	2,16	24	2203,79	2666,25

[1] % fixed: the percentage of simulations where an adaptive variant arose and fixed in the population

[2] Mean gen. to fixation: the mean generations for the variant to fix after arising

[3] % not fixed: the percentage of simulations where a potentially adaptive variant arose but did not fix

[4] Mean gen. to loss: mean number of generations for a potentially adaptive variant to be lost, given that it is lost

[5] % no mutations: the percentage of simulations where no potentially adaptive mutation arose

[6] Mean gen. to 1st segregating potentially adaptive variant: mean number of generations for the first potentially adaptive variant to occur

[7] Mean gen to adapt: the mean number of generations for an adaptive variant to fix given that adaptation occurred, note that this differs from the sum of columns H and D because in some cases potentially adaptive mutations are lost before an adaptive variant eventually fixes.

Supplementary Table 9. Primers used in this study.

OligoName	OligoSequence
FLC_RqPCR1_F	CCGAACTCATGTTGAAGCTTGTTGAG
FLC_RqPCR1_R	CGGAGATTTGTCCAGCAGGTG
PP2A_qPCR2_F	AAATACGCCCAACGAACAAA
PP2A_qPCR2_R	CAGCAACGAATTGTGTTTGG
Oligo(dT)	TTTTTTTTTTTTTTTTTT

Supplementary Table 10. Coalescence rate and effective population size (Ne) over time inferred in RELATE for FRI 232X in Santo Antão and with the CVI-Ancestor individual, used with the --coal argument in CLUES.

time	haploid.coalescence.rate	pop_size
0	1,61E-04	3113,65462
1000	2,48E-04	2019,43504
1389,5	6,70E-04	745,886807
1930,7	8,03E-04	622,579721
2682,7	6,21E-05	8048,17315
3727,59	1,16E-05	43164,8465
5179,48	0,00E+00	Inf
7196,86	0,00E+00	Inf
10000	2,60E-06	192187,206
13895	1,06E-05	46969,0849
19307	0,00E+00	Inf
26827	4,67E-04	1069,83681
37275,9	4,67E-04	1069,83681
51794,8	4,67E-04	1069,83681
71968,6	4,67E-04	1069,83681
1,00E+05	4,67E-04	1069,83681
138950	4,67E-04	1069,83681
193070	4,67E-04	1069,83681
268270	4,67E-04	1069,83681
372759	4,67E-04	1069,83681
517948	4,67E-04	1069,83681
719686	4,67E-04	1069,83681
1,00E+06	4,67E-04	1069,83681
1389500	4,67E-04	1069,83681
1930700	4,67E-04	1069,83681
2682700	4,67E-04	1069,83681
3727590	4,67E-04	1069,83681
5179480	4,67E-04	1069,83681
7196860	4,67E-04	1069,83681
1,00E+07	4,67E-04	1069,83681
1,00E+07	4,67E-04	1069,83681

Supplementary Table 11. Coalescence rate and effective population size (Ne) over time inferred from genome-wide data in RELATE and used with the --coal argument in CLUES.

time	haploid.coalescence.rate	pop_size
0	1,92E-04	2604,0446
1000	3,88E-05	12870,046
1389,5	1,07E-04	4662,74374
1930,7	2,30E-04	2177,89955
2682,7	1,17E-03	427,785525
3727,59	1,18E-03	424,358158
5179,48	1,36E-03	367,395825
7196,86	1,07E-01	4,68239326
10000	1,07E-01	4,68239326
13895	1,07E-01	4,68239326
19307	1,07E-01	4,68239326
26827	1,07E-01	4,68239326
37275,9	1,07E-01	4,68239326
51794,8	1,07E-01	4,68239326
71968,6	1,07E-01	4,68239326
1,00E+05	1,07E-01	4,68239326
138950	1,07E-01	4,68239326
193070	1,07E-01	4,68239326
268270	1,07E-01	4,68239326
372759	1,07E-01	4,68239326
517948	1,07E-01	4,68239326
719686	1,07E-01	4,68239326
1,00E+06	1,07E-01	4,68239326
1389500	1,07E-01	4,68239326
1930700	1,07E-01	4,68239326
2682700	1,07E-01	4,68239326
3727590	1,07E-01	4,68239326
5179480	1,07E-01	4,68239326
7196860	1,07E-01	4,68239326
1,00E+07	1,07E-01	4,68239326
1,00E+07	1,07E-01	4,68239326

Supplementary References

1. Fick, S. E. & Hijmans, R. J. WorldClim2: new 1-km spatial resolution climate surfaces for global land areas. *Int. J. Climatol.* **37**, 4302–4315 (2017).
2. Antonio Trabucco & Robert J. Zomer. Global aridity index and potential evapo-transpiration (ET0) climate database v2. (2019).
3. Lasky, J. R. *et al.* Characterizing genomic variation of *Arabidopsis thaliana*: the roles of geography and climate. *Mol. Ecol.* **21**, 5512–5529 (2012).
4. Durvasula, A. *et al.* African genomes illuminate the early history and transition to selfing in *Arabidopsis thaliana*. *Proc Natl Acad Sci USA* **114**, 5213 (2017).
5. Ossowski, S. *et al.* Sequencing of natural strains of *Arabidopsis thaliana* with short reads. *Genome Res.* **18**, 2024–2033 (2008).
6. Li, H. & Durbin, R. Fast and accurate short read alignment with Burrows-Wheeler transform. *Bioinforma. Oxf. Engl.* **25**, 1754–1760 (2009).
7. Schubert, M., Lindgreen, S. & Orlando, L. AdapterRemoval v2: rapid adapter trimming, identification, and read merging. *BMC Res. Notes* **9**, 88 (2016).
8. McKenna, A. *et al.* The genome analysis toolkit: A MapReduce framework for analyzing next-generation DNA sequencing data. *Genome Res.* **20**, 1297–1303 (2010).
9. Li, H. *et al.* The sequence alignment/Map format and SAMtools. *Bioinformatics* **25**, 2078–2079 (2009).

10. Danecek, P. *et al.* Twelve years of SAMtools and BCFtools. *GigaScience* **10**, giab008 (2021).

11. Danecek, P. *et al.* The variant call format and VCFtools. *Bioinformatics* **27**, 2156–2158 (2011).

12. Tang, C. *et al.* The evolution of selfing in *Arabidopsis thaliana*. *Science* **317**, 1070–1072 (2007).

13. Dwyer, K. G. *et al.* Molecular characterization and evolution of self-incompatibility genes in *Arabidopsis thaliana*: the case of the Sc haplotype. *Genetics* **193**, 985–994 (2013).

14. Salomé, P. A. *et al.* Genetic architecture of flowering-time variation in *Arabidopsis thaliana*. *Genetics* **188**, 421–433 (2011).

15. Purcell, S. *et al.* PLINK: a tool set for whole-genome association and population-based linkage analyses. *Am J Hum Genet* **81**, 559–575 (2007).

16. Schiffels, S. & Durbin, R. Inferring human population size and separation history from multiple genome sequences. *Nat Genet* **46**, 919–925 (2014).

17. Gutenkunst, R. N., Hernandez, R. D., Williamson, S. H. & Bustamante, C. D. Inferring the joint demographic history of multiple populations from multidimensional SNP frequency data. *PLOS Genet.* **5**, e1000695 (2009).

18. Kelleher, J., Etheridge, A. M. & McVean, G. Efficient coalescent simulation and genealogical analysis for large sample sizes. *PLOS Comput. Biol.* **12**, e1004842 (2016).

19. Novikova, P. Y. *et al.* Sequencing of the genus *Arabidopsis* identifies a complex history of nonbifurcating speciation and abundant trans-specific polymorphism. *Nat. Genet.* **48**, 1077–1082 (2016).

20. Stamatakis, A. RAxML version 8: a tool for phylogenetic analysis and post-analysis of large phylogenies. *Bioinforma. Oxf. Engl.* **30**, 1312–1313 (2014).

21. Drummond, A. J., Suchard, M. A., Xie, D. & Rambaut, A. Bayesian phylogenetics with BEAUti and the BEAST 1.7. *Mol. Biol. Evol.* **29**, 1969–1973 (2012).

22. Hohmann, N., Wolf, E. M., Lysak, M. A. & Koch, M. A. A time-calibrated road map of Brassicaceae species radiation and evolutionary history. *Plant Cell* **27**, 2770–2784 (2015).

23. Drummond, A. J., Ho, S. Y. W., Phillips, M. J. & Rambaut, A. Relaxed phylogenetics and dating with confidence. *PLoS Biol.* **4**, e88 (2006).

24. Gernhard, T. The conditioned reconstructed process. *J. Theor. Biol.* **253**, 769–778 (2008).

25. Haller, B. C. & Messer, P. W. SLiM 3: forward genetic simulations beyond the Wright-Fisher model. *Mol Biol Evol* **36**, 632–637 (2019).

26. Phillips, S. J., Anderson, R. P. & Schapire, R. E. Maximum entropy modeling of species geographic distributions. *Ecol. Model.* **190**, 231–259 (2006).

27. Brennan, A. C. *et al.* The genetic structure of *Arabidopsis thaliana* in the south-western Mediterranean range reveals a shared history between North Africa and southern Europe. *BMC Plant Biol.* **14**, 17 (2014).

28. Cingolani, P. *et al.* A program for annotating and predicting the effects of single nucleotide polymorphisms, SnpEff. *Fly (Austin)* **6**, 80–92 (2012).

29. Tataru, P. & Bataillon, T. polyDFE: inferring the distribution of fitness effects and properties of beneficial mutations from polymorphism data. *Methods Mol Biol* **2090**, 125–146 (2020).

30. W. Lobin. The occurrance of *Arabidopsis thaliana* in the Cape Verde Islands. in vol. 20 119–123 (1983).

31. Alonso-Blanco, C. *et al.* Development of an AFLP based linkage map of Ler, Col and Cvi *Arabidopsis thaliana* ecotypes and construction of a Ler/Cvi recombinant inbred line population: AFLP based linkage map of *Arabidopsis*. *Plant J.* **14**, 259–271 (1998).

32. Simon, M. *et al.* Quantitative trait loci mapping in five new large recombinant inbred line populations of *Arabidopsis thaliana* genotyped with consensus single-nucleotide polymorphism markers. *Genetics* **178**, 2253–2264 (2008).

33. Keurentjes, J. J. B. *et al.* Development of a near-isogenic line population of *Arabidopsis thaliana* and comparison of mapping power with a recombinant inbred line population. *Genetics* **175**, 891–905 (2007).

34. Alonso-Blanco, C., El-Assal, S. E., Coupland, G. & Koornneef, M. Analysis of natural allelic variation at flowering time loci in the Landsberg erecta and Cape Verde Islands ecotypes of *Arabidopsis thaliana*. *Genetics* **149**, 749–764 (1998).

35. Alonso-Blanco, C. *et al.* Genetic and molecular analyses of natural variation indicate CBF2 as a candidate gene for underlying a freezing tolerance quantitative trait locus in *Arabidopsis*. *Plant Physiol* **139**, 1304–1312 (2005).

36. Alonso-Blanco, C., Vries, H. B., Hanhart, C. J. & Koornneef, M. Natural allelic variation at seed size loci in relation to other life history traits of *Arabidopsis thaliana*. *PNAS* **96**, 4710–4717 (1999).

37. Bandaranayake, C. K., Koumproglou, R., Wang, X. Y., Wilkes, T. & Kearsey, M. J. QTL analysis of morphological and developmental traits in the Ler × Cvi population of *Arabidopsis thaliana*. *Euphytica* **137**, 361–371 (2004).

38. Bentsink, L. *et al.* Genetic analysis of seed-soluble oligosaccharides in relation to seed storability of *Arabidopsis*. *Plant Physiol.* **124**, 1595–1604 (2000).

39. Bentsink, L. *et al.* Natural variation for seed dormancy in *Arabidopsis* is regulated by additive genetic and molecular pathways. *PNAS* **107**, 4264–4269 (2010).

40. Bentsink, L., Yuan, K., Koornneef, M. & Vreugdenhil, D. The genetics of phytate and phosphate accumulation in seeds and leaves of *Arabidopsis thaliana*, using natural variation. *Theor Appl Genet* **106**, 1234–1243 (2003).

41. Borevitz, J. O. *et al.* Quantitative trait loci controlling light and hormone response in two accessions of *Arabidopsis thaliana*. *Genetics* **160**, 683–696 (2002).

42. Botto, J. F., Alonso-Blanco, C., Garzarón, I., Sánchez, R. A. & Casal, J. J. The Cape Verde Islands allele of Cryptochrome 2 enhances cotyledon unfolding in the absence of blue light in *Arabidopsis*. *Plant Physiol.* **133**, 1547–1556 (2003).

43. Buescher, E. *et al.* Natural genetic variation in selected populations of *Arabidopsis thaliana* is associated with ionomic differences. *PLOS ONE* **5**, e11081 (2010).

44. Coneva, V. & Chitwood, D. H. Genetic and developmental basis for increased leaf thickness in the *Arabidopsis* Cvi ecotype. *Front Plant Sci* **9**, (2018).

45. Conte, M., de Simone, S., Simmons, S. J., Ballaré, C. L. & Stapleton, A. E. Chromosomal loci important for cotyledon opening under UV-B in *Arabidopsis thaliana*. *BMC Plant Biol.* **10**, 112 (2010).

46. Darrah, C. *et al.* Analysis of phase of LUCIFERASE expression reveals novel circadian quantitative trait loci in *Arabidopsis*. *Plant Physiol.* **140**, 1464–1474 (2006).

47. DeRose-Wilson, L. & Gaut, B. S. Mapping salinity tolerance during *Arabidopsis thaliana* germination and seedling growth. *PLOS ONE* **6**, e22832 (2011).

48. Edwards, K. D., Lynn, J. R., Gyula, P., Nagy, F. & Millar, A. J. Natural allelic variation in the temperature-compensation mechanisms of the *Arabidopsis thaliana* circadian clock. *Genetics* **170**, 387–400 (2005).

49. Fournier-Level, A. *et al.* Paths to selection on life history loci in different natural environments across the native range of *Arabidopsis thaliana*. *Mol Ecol* **22**, 3552–3566 (2013).

50. Gilliland, L. U. *et al.* Genetic basis for natural variation in seed vitamin E levels in *Arabidopsis thaliana*. *PNAS* **103**, 18834–18841 (2006).

51. Hobbs, D. H., Flintham, J. E. & Hills, M. J. Genetic control of storage oil synthesis in seeds of *Arabidopsis*. *Plant Physiol.* **136**, 3341–3349 (2004).

52. Kasulin, L., Agrofoglio, Y. & Botto, J. F. The receptor-like kinase ERECTA contributes to the shade-avoidance syndrome in a background-dependent manner. *Ann Bot* **111**, 811–819 (2013).

53. Keurentjes, J. J. *et al.* Integrative analyses of genetic variation in enzyme activities of primary carbohydrate metabolism reveal distinct modes of regulation in *Arabidopsis thaliana*. *Genome Biol.* **9**, R129 (2008).

54. Kliebenstein, D. J., Gershenzon, J. & Mitchell-Olds, T. Comparative quantitative trait loci mapping of aliphatic, indolic and benzylic glucosinolate production in *Arabidopsis thaliana* leaves and seeds. *Genetics* **159**, 359–370 (2001).

55. Kliebenstein, D., Pedersen, D., Barker, B. & Mitchell-Olds, T. Comparative analysis of quantitative trait loci controlling glucosinolates, myrosinase and insect resistance in *Arabidopsis thaliana*. *Genetics* **161**, 325–332 (2002).

56. Kobayashi, Y. *et al.* Amino acid polymorphisms in strictly conserved domains of a P-type ATPase HMA5 are involved in the mechanism of copper tolerance variation in *Arabidopsis*. *Plant Physiol* **148**, 969–980 (2008).

57. Laserna, M. P., Sánchez, R. A. & Botto, J. F. Light-related loci controlling seed germination in Ler x Cvi and Bay-0 x Sha recombinant inbred-line populations of *Arabidopsis thaliana*. *Ann Bot* **102**, 631–642 (2008).

58. Lee, S., Sergeeva, L. I. & Vreugdenhil, D. Quantitative trait loci analysis of hormone levels in *Arabidopsis* roots. *PLOS ONE* **14**, e0219008 (2019).

59. Li, P. *et al.* Fructose sensitivity is suppressed in *Arabidopsis* by the transcription factor ANAC089 lacking the membrane-bound domain. *PNAS* **108**, 3436–3441 (2011).

60. Luquez, V. M. C. *et al.* Quantitative trait loci analysis of leaf and plant longevity in *Arabidopsis thaliana*. *J Exp Bot* **57**, 1363–1372 (2006).

61. Moore, C. R., Gronwall, D. S., Miller, N. D. & Spalding, E. P. Mapping quantitative trait loci affecting *Arabidopsis thaliana* seed morphology features extracted computationally from images. *G3 Bethesda* **3**, 109–118 (2013).

62. Nguyen, T.-P., Keizer, P., Eeuwijk, F. van, Smeekens, S. & Bentsink, L. Natural variation for seed longevity and seed dormancy are negatively correlated in *Arabidopsis*. *Plant Physiol.* **160**, 2083–2092 (2012).

63. Sergeeva, L. I. *et al.* Histochemical analysis reveals organ-specific quantitative trait loci for enzyme activities in *Arabidopsis*. *Plant Physiol* **134**, 237–245 (2004).

64. Sergeeva, L. I. *et al.* Vacuolar invertase regulates elongation of *Arabidopsis thaliana* roots as revealed by QTL and mutant analysis. *PNAS* **103**, 2994–2999 (2006).

65. Snoek, B. L. *et al.* Genetic dissection of morphometric traits reveals that Phytochrome B affects nucleus size and heterochromatin organization in *Arabidopsis thaliana*. *G3 Bethesda* **7**, 2519–2531 (2017).

66. Swarup, K. *et al.* Natural allelic variation identifies new genes in the *Arabidopsis* circadian system. *Plant J.* **20**, 67–77 (1999).

67. Symonds, V. V. *et al.* Mapping quantitative trait loci in multiple populations of *Arabidopsis thaliana* identifies natural allelic Variation for trichome density. *Genetics* **169**, 1649–1658 (2005).

68. Teng, S., Keurentjes, J., Bentsink, L., Koornneef, M. & Smeekens, S. Sucrose-specific induction of anthocyanin biosynthesis in *Arabidopsis* requires the MYB75/PAP1 gene. *Plant Physiol.* **139**, 1840–1852 (2005).

69. Tessadori, F. *et al.* PHYTOCHROME B and HISTONE DEACETYLASE 6 control light-induced chromatin compaction in *Arabidopsis thaliana*. *PLOS Genet.* **5**, e1000638 (2009).

70. Ungerer, M. C., Halldorsdottir, S. S., Modliszewski, J. L., Mackay, T. F. C. & Purugganan, M. D. Quantitative trait loci for inflorescence development in *Arabidopsis thaliana*. *Genetics* **160**, 1133–1151 (2002).

71. Vasseur, F., Bontpart, T., Dauzat, M., Granier, C. & Vile, D. Multivariate genetic analysis of plant responses to water deficit and high temperature revealed contrasting adaptive strategies. *J Exp Bot* **65**, 6457–6469 (2014).

72. Vaughn, L. M. & Masson, P. H. A QTL study for regions contributing to *Arabidopsis thaliana* root skewing on tilted surfaces. *G3 Genes Genomes Genet.* **1**, 105–115 (2011).

73. Velázquez, I., Valencia, S., López-Lera, A., de la Peña, A. & Candela, M. Analysis of natural allelic variation in in vitro organogenesis of *Arabidopsis thaliana*. *Euphytica* **137**, 73–79 (2004).

74. Ward, J. K. *et al.* Identification of a major QTL that alters flowering time at elevated [CO2] in *Arabidopsis thaliana*. *PLOS ONE* **7**, e49028 (2012).

75. Waters, B. M. & Grusak, M. A. Quantitative trait locus mapping for seed mineral concentrations in two *Arabidopsis thaliana* recombinant inbred populations. *New Phytol.* **179**, 1033–1047 (2008).

76. Juenger, T. E. *et al.* Identification and characterization of QTL underlying whole-plant physiology in *Arabidopsis thaliana*: δ13C, stomatal conductance and transpiration efficiency. *Plant Cell Environ.* **28**, 697–708 (2005).

77. Juenger, T., Pérez-Pérez, J. M., Bernal, S. & Micol, J. L. Quantitative trait loci mapping of floral and leaf morphology traits in *Arabidopsis thaliana*: evidence for modular genetic architecture. *Evol Dev* **7**, 259–271 (2005).

78. Aranzana, M. J. *et al.* Genome-wide association mapping in *Arabidopsis* identifies previously known flowering time and pathogen resistance genes. *PLoS Genet* **1**, (2005).

79. Bikard, D. *et al.* Divergent evolution of duplicate genes leads to genetic incompatibilities within *A. thaliana*. *Science* **323**, 623–626 (2009).

80. Brachi, B. *et al.* Linkage and association mapping of *Arabidopsis thaliana* flowering time in nature. *PLOS Genet.* **6**, e1000940 (2010).

81. Dunning, F. M., Sun, W., Jansen, K. L., Helft, L. & Bent, A. F. Identification and mutational analysis of *Arabidopsis* FLS2 leucine-rich repeat domain residues that contribute to flagellin perception. *Plant Cell* **19**, 3297–3313 (2007).

82. Egli, B., Kölling, K., Köhler, C., Zeeman, S. C. & Streb, S. Loss of cytosolic phosphoglucomutase compromises gametophyte development in *Arabidopsis*. *Plant Physiol.* **154**, 1659–1671 (2010).

83. Ehrenreich, I. M., Stafford, P. A. & Purugganan, M. D. The genetic architecture of shoot branching in *Arabidopsis thaliana*: A comparative assessment of candidate gene associations vs. quantitative trait locus mapping. *Genetics* **176**, 1223–1236 (2007).

84. El-Din El-Assal, S., Alonso-Blanco, C., Peeters, A. J. M., Raz, V. & Koornneef, M. A QTL for flowering time in *Arabidopsis* reveals a novel allele of CRY2. *Nat. Genet.* **29**, 435–440 (2001).

85. Fulcher, N. *et al.* Genetic architecture of natural variation of telomere length in *Arabidopsis thaliana*. *Genetics* **199**, 625–635 (2015).

86. Gazzani, S., Gendall, A. R., Lister, C. & Dean, C. Analysis of the molecular basis of flowering time variation in *Arabidopsis* accessions. *Plant Physiol* **132**, 1107–1114 (2003).

87. Kadirjan-Kalbach, D. K. *et al.* Allelic variation in the chloroplast division gene FtsZ2-2 leads to natural variation in chloroplast size. *Plant Physiol.* **181**, 1059–1074 (2019).

88. Kim, T.-S., Wang, L., Kim, Y. J. & Somers, D. E. Compensatory mutations in GI and ZTL may modulate temperature compensation in the circadian clock. *Plant Physiol.* **182**, 1130–1141 (2020).

89. Kooke, R. *et al.* Genome-wide association mapping and genomic prediction elucidate the genetic architecture of morphological traits in *Arabidopsis*. *Plant Physiol.* **170**, 2187–2203 (2016).

90. Leskow, C. C. *et al.* Allelic differences in a vacuolar invertase affect *Arabidopsis* growth at early plant development. *J Exp Bot* **67**, 4091–4103 (2016).

91. Li, W. *et al.* Identification of quantitative trait loci controlling high calcium response in *Arabidopsis thaliana*. *PLOS ONE* **9**, e112511 (2014).

92. Marais, D. L. D. *et al.* Variation in MPK12 affects water use efficiency in *Arabidopsis* and reveals a pleiotropic link between guard cell size and ABA response. *PNAS* **111**, 2836–2841 (2014).

93. Ouibrahim, L. *et al.* Cloning of the *Arabidopsis* rwm1 gene for resistance to Watermelon mosaic virus points to a new function for natural virus resistance genes. *Plant J* **79**, 705–716 (2014).

94. Routaboul, J.-M. *et al.* Metabolite profiling and quantitative genetics of natural variation for flavonoids in *Arabidopsis*. *J Exp Bot* **63**, 3749–3764 (2012).

95. Seedat, N., Dinsdale, A., Ong, E. K. & Gendall, A. R. Acceleration of flowering in *Arabidopsis thaliana* by Cape Verde Islands alleles of FLOWERING H is dependent on the floral promoter FD. *J Exp Bot* **64**, 2767–2778 (2013).

96. Yano, R., Takebayashi, Y., Nambara, E., Kamiya, Y. & Seo, M. Combining association mapping and transcriptomics identify HD2B histone deacetylase as a genetic factor associated with seed dormancy in *Arabidopsis thaliana*. *Plant J* **74**, 815–828 (2013).

97. Yuan, W., Flowers, J. M., Sahraie, D. J. & Purugganan, M. D. Cryptic genetic variation for *Arabidopsis thaliana* seed germination speed in a novel salt stress environment. *G3 Genes Genomes Genet.* **6**, 3129–3138 (2016).

98. Alonso-Blanco, C., Bentsink, L., Hanhart, C. J., Vries, H. B. & Koornneef, M. Analysis of natural allelic variation at seed dormancy loci of *Arabidopsis thaliana*. *Genetics* **164**, 711–729 (2003).

99. Moore, C. R. *et al.* High-throughput computer vision introduces the time axis to a quantitative trait map of a plant growth response. *Genetics* **195**, 1077–1086 (2013).

100. O'Neill, C. M. *et al.* Towards the genetic architecture of seed lipid biosynthesis and accumulation in *Arabidopsis thaliana*. *Heredity* **108**, 115–123 (2012).

101. Nguyen, T.-P., Cueff, G., Hegedus, D. D., Rajjou, L. & Bentsink, L. A role for seed storage proteins in *Arabidopsis* seed longevity. *J Exp Bot* **66**, 6399–6413 (2015).

102. Keurentjes, J. J. B. *et al.* Regulatory network construction in *Arabidopsis* by using genome-wide gene expression quantitative trait loci. *PNAS* **104**, 1708–1713 (2007).

103. McKhann, H. I. *et al.* Natural variation in CBF gene sequence, gene expression and freezing tolerance in the Versailles core collection of *Arabidopsis thaliana*. *BMC Plant Biol.* **8**, 105 (2008).

104. Poque, S. *et al.* Allelic variation at the rpv1 locus controls partial resistance to *Plum pox* virus infection in *Arabidopsis thaliana*. *BMC Plant Biol.* **15**, 159 (2015).

105. Kuhn, M. Building predictive models in R using the caret package. *J. Stat. Softw.* **28**, (2008).

106. Signorell, A. *DescTools: tools for descriptive statistics*. (2020).

107. Fisher, R. A. Statistical methods for research workers. in *Breakthroughs in Statistics: Methodology and Distribution* (eds. Kotz, S. & Johnson, N. L.) 66–70 (Springer, 1992). doi:10.1007/978-1-4612-4380-9_6.

108. Moulos, P. & Hatzis, P. Systematic integration of RNA-Seq statistical algorithms for accurate detection of differential gene expression patterns. *Nucleic Acids Res* **43**, e25–e25 (2015).

109. Shindo, C. *et al.* Role of FRIGIDA and FLOWERING LOCUS C in determining variation in flowering time of *Arabidopsis*. *Plant Physiol* **138**, 1163 (2005).

110. Werner, J. D. *et al.* FRIGIDA-independent variation in flowering time of natural *Arabidopsis thaliana* accessions. *Genetics* **170**, 1197–1207 (2005).

111. Michaels, S. D. & Amasino, R. M. FLOWERING LOCUS C encodes a novel MADS domain protein that acts as a repressor of flowering. *Plant Cell* **11**, 949 (1999).

112. Mendiburu, F. Agricolae: statistical procedures for agricultural research. *R Package Version* **1**, 1–8 (2010).

113. Speidel, L., Forest, M., Shi, S. & Myers, S. R. A method for genome-wide genealogy estimation for thousands of samples. *Nat. Genet.* **51**, 1321–1329 (2019).

114. Li, H. A statistical framework for SNP calling, mutation discovery, association mapping and population genetical parameter estimation from sequencing data. *Bioinformatics* **27**, 2987–2993 (2011).

115. Ossowski, S. *et al.* The rate and molecular spectrum of spontaneous mutations in *Arabidopsis thaliana*. *Science* **327**, 92–94 (2010).

116. Salomé, P. A. *et al.* The recombination landscape in *Arabidopsis thaliana* F2 populations. *Hered. Edinb* **108**, 447–455 (2012).

117. Bomblies, K. *et al.* Local-scale patterns of genetic variability, outcrossing, and spatial structure in natural stands of *Arabidopsis thaliana*. *PLoS Genet* **6**, e1000890 (2010).

118. Speidel, L. *et al.* Inferring population histories for ancient genomes using genome-wide genealogies. *Mol. Biol. Evol.* (2021) doi:10.1093/molbev/msab174.

119. Stern, A. J., Wilton, P. R. & Nielsen, R. An approximate full-likelihood method for inferring selection and allele frequency trajectories from DNA sequence data. *PLOS Genet.* **15**, e1008384 (2019).

120. Gillespie, J. H. Some properties of finite populations experiencing strong selection and weak mutation. *Am. Nat.* **121**, 691–708 (1983).

121. Gillespie, J. H. Molecular evolution over the mutational landscape. *Evolution* **38**, 1116–1129 (1984).

122. Gillespie, J. H. *The causes of molecular evolution*. (Oxford University Press, 1991).

123. Zhang, L. & Jiménez-Gómez, J. M. Functional analysis of FRIGIDA using naturally occurring variation in *Arabidopsis thaliana*. *Plant J.* **103**, 154–165 (2020).

124. Sheldon, C. C., Conn, A. B., Dennis, E. S. & Peacock, W. J. Different regulatory regions are required for the vernalization-induced repression of FLOWERING LOCUS C and for the epigenetic maintenance of repression. *Plant Cell* **14**, 2527–2537 (2002).

125. Caicedo, A. L., Stinchcombe, J. R., Olsen, K. M., Schmitt, J. & Purugganan, M. D. Epistatic interaction between *Arabidopsis* FRI and FLC flowering time genes generates a latitudinal cline in a life history trait. *Proc Natl Acad Sci USA* **101**, 15670–15675 (2004).

126. Sung, S. *et al.* Epigenetic maintenance of the vernalized state in *Arabidopsis thaliana* requires LIKE HETEROCHROMATIN PROTEIN 1. *Nat. Genet.* **38**, 706–710 (2006).

127. Lawson, D. J., Hellenthal, G., Myers, S. & Falush, D. Inference of population structure using dense haplotype data. *PLoS Genet* **8**, e1002453 (2012).

128. Alonso-Blanco, C. *et al.* 1,135 genomes reveal the global pattern of polymorphism in *Arabidopsis thaliana*. *Cell* **166**, 481–491 (2016).

129. Boggs, N. A., Nasrallah, J. B. & Nasrallah, M. E. Independent S-locus mutations caused self-fertility in *Arabidopsis thaliana*. *PLOS Genet.* **5**, e1000426 (2009).

130. Sherman-Broyles, S. *et al.* S locus genes and the evolution of self-fertility in *Arabidopsis thaliana*. *Plant Cell* **19**, 94–106 (2007).

131. Shimizu, K. K., Shimizu-Inatsugi, R., Tsuchimatsu, T. & Purugganan, M. D. Independent origins of self-compatibility in *Arabidopsis thaliana*. *Mol. Ecol.* **17**, 704–714 (2008).

132. Shimizu, K. K. & Tsuchimatsu, T. Evolution of selfing: recurrent patterns in molecular adaptation. *Annu. Rev. Ecol. Evol. Syst.* **46**, 593–622 (2015).

133. Tsuchimatsu, T. *et al.* Patterns of polymorphism at the self-incompatibility locus in 1,083 *Arabidopsis thaliana* genomes. *Mol. Biol. Evol.* **34**, 1878–1889 (2017).

134. Schiffels, S. & Wang, K. MSMC and MSMC2: the multiple sequentially Markovian coalescent. *Methods Mol Biol* **2090**, 147–166 (2020).

135. Wright, S. Isolation by Distance. *Genetics* **28**, 114–138 (1943).

136. Malécot, M. Les mathématiques de l’hérédité. *Bull. Mens. Société Linn. Lyon* 203 (1948).

137. Loveless, M. D. & Hamrick, J. L. Ecological determinants of genetic structure in plant populations. *Annu. Rev. Ecol. Syst.* **15**, 65–95 (1984).

138. Schmid, K. J. *et al.* Evidence for a large-scale population structure of *Arabidopsis thaliana* from genome-wide single nucleotide polymorphism markers. *Theor. Appl. Genet.* **112**, 1104–1114 (2006).

139. Platt, A. *et al.* The scale of population structure in *Arabidopsis thaliana*. *PLOS Genet.* **6**, e1000843 (2010).

140. Nordborg, M. *et al.* The pattern of polymorphism in *Arabidopsis thaliana*. *PLOS Biol.* **3**, e196 (2005).

141. Horton, M. W. *et al.* Genome-wide patterns of genetic variation in worldwide *Arabidopsis thaliana* accessions from the RegMap panel. *Nat. Genet.* **44**, 212–216 (2012).

142. Zhou, X. & Stephens, M. Genome-wide efficient mixed-model analysis for association studies. *Nat Genet* **44**, 821–824 (2012).

143. Coupland, G. FLOWERING LOCUS C isolation and characterization: two articles that opened many doors. *Plant Cell* **31**, 1190 (2019).

144. Salathia, N. *et al.* FLOWERING LOCUS C -dependent and -independent regulation of the circadian clock by the autonomous and vernalization pathways. *BMC Plant Biol* **6**, 10 (2006).

145. Osmond, M. M., Otto, S. P. & Martin, G. Genetic paths to evolutionary rescue and the distribution of fitness effects along them. *Genetics* **214**, 493–510 (2020).

146. Szendro, I. G., Franke, J., de Visser, J. A. G. M. & Krug, J. Predictability of evolution depends nonmonotonically on population size. *Proc. Natl. Acad. Sci.* **110**, 571–576 (2013).

147. Orr, H. A. Theories of adaptation: what they do and don't say. *Genetica* **123**, 3–13 (2005).

148. Orr, H. A. The population genetics of adaptation: the adaptation of DNA sequences. *Evolution* **56**, 1317–1330 (2002).

149. Barghi, N., Hermisson, J. & Schlötterer, C. Polygenic adaptation: a unifying framework to understand positive selection. *Nat. Rev. Genet.* 1–13 (2020) doi:10.1038/s41576-020-0250-z.

150. Höllinger, I., Pennings, P. S. & Hermisson, J. Polygenic adaptation: From sweeps to subtle frequency shifts. *PLOS Genet.* **15**, e1008035 (2019).

151. Cho, L.-H., Yoon, J. & An, G. The control of flowering time by environmental factors. *Plant J.* **90**, 708–719 (2017).

152. Bloomer, R. H. & Dean, C. Fine-tuning timing: natural variation informs the mechanistic basis of the switch to flowering in *Arabidopsis thaliana*. *J. Exp. Bot.* **68**, 5439–5452 (2017).

153. Mouradov, A., Cremer, F. & Coupland, G. Control of flowering time: interacting pathways as a basis for diversity. *Plant Cell* **14**, S111–S130 (2002).

154. He, Y., Chen, T. & Zeng, X. Genetic and epigenetic understanding of the seasonal timing of flowering. *Plant Commun.* **1**, (2020).

155. Korkuć, P., Schippers, J. H. M. & Walther, D. Characterization and identification of cis-regulatory elements in *Arabidopsis* based on single-nucleotide polymorphism information. *Plant Physiol.* **164**, 181–200 (2014).

156. Johanson, U. *et al.* Molecular analysis of FRIGIDA, a major determinant of natural variation in *Arabidopsis* flowering time. *Science* **290**, 344–347 (2000).

157. Schläppi, M. RNA levels and activity of FLOWERING LOCUS C are modified in mixed genetic backgrounds of *Arabidopsis thaliana*. *Int. J. Plant Sci.* **162**, 527–537 (2001).

158. Le Corre, V., Roux, F. & Reboud, X. DNA polymorphism at the FRIGIDA gene in *Arabidopsis thaliana*: extensive nonsynonymous variation is consistent with local selection for flowering time. *Mol Biol Evol* **19**, 1261–1271 (2002).

159. Michaels, S. D., He, Y., Scortecci, K. C. & Amasino, R. M. Attenuation of FLOWERING LOCUS C activity as a mechanism for the evolution of summer-annual flowering behavior in *Arabidopsis*. *Proc. Natl. Acad. Sci.* **100**, 10102–10107 (2003).

160. Google Earth. Santo Antão: 17 degrees 10’N 25 degrees 05’ W to 14 degrees 55’N 24 degrees 17’W.

161. Google Earth. Fogo: 15 degrees 10’ N 24 degrees 25’ W, 14 degrees 55’ N 24 degrees 17’ W.

162. Esri. World Imagery 1:5x01^7. (2009).

163. Graul, C. *Interactive Web-Maps Based on the Leaflet JavaScript Library*. (2016).
