# Supplementary material for: Parallel reduction in flowering time from de novo mutations enable evolutionary rescue in colonizing lineages
Source: Nat Commun. 2022 Mar 18;13:1461. doi: 10.1038/s41467-022-28800-z (PMC8933414; doi:10.1038/s41467-022-28800-z)
Supplement: Supplementary file 3 — Description of Additional Supplementary Files [file 41467_2022_28800_MOESM3_ESM.pdf]

Description of Additional Supplementary Files

File Name: Supplementary Data 1
Description: Accessions sequenced in this study. For each accession, sequencing ID, sample ID, geographic cluster and population, latitude and longitude, island of origin, and sequencing coverage are shown.

File Name: Supplementary Data 2
Description: Differences in climatic variable distributions between Cape Verde, Morocco and Eurasia. P-values and W statistic are shown per variable and calculated using Wilcoxon test. Aridity index is classified in hyper arid (< 0.03), arid (0.03 – 0.2), semi-arid (0.2 – 0.5), dry sub-humid (0.5 – 0.65) and humid (> 0.65), according to UNEP 1997.

File Name: Supplementary Data 3
Description: Correlation matrix between climate variables in Morocco. Values show Pearson's R. Climatic variables are the same as in Supplementary Data 2.

File Name: Supplementary Data 4
Description: Fitness differences between populations and conditions. Results shown for negative binomial GLM. The Moroccan population under the CVI simulated conditions was used as the baseline for the model, so all comparisons are made against it.

File Name: Supplementary Dataset 5
Description: QTL identified in studies with Cvi-0.

File Name: Supplementary Data 6
Description: Candidate variants from bulk segregant analysis, with genomic position, alternative and reference alleles, predicted impact using SnpEff, amino acid change, affected gene and frequency in Fogo shown.
