# Supplementary material for: Parallel reduction in flowering time from de novo mutations enable evolutionary rescue in colonizing lineages
Source: Nat Commun. 2022 Mar 18;13:1461. doi: 10.1038/s41467-022-28800-z (PMC8933414; doi:10.1038/s41467-022-28800-z)
Supplement: Supplementary file 10 — Reporting Summary [file 41467_2022_28800_MOESM10_ESM.pdf]

nature research

Corresponding author(s): Angela M Hancock

Last updated by author(s): 02/04/2022

Reporting Summary

Nature Research wishes to improve the reproducibility of the work that we publish. This form provides structure for consistency and transparency in reporting. For further information on Nature Research policies, see our Editorial Policies and the Editorial Policy Checklist.

Statistics

For all statistical analyses, confirm that the following items are present in the figure legend, table legend, main text, or Methods section.

n/a	Confirmed	
☐	☒	The exact sample size (*n*) for each experimental group/condition, given as a discrete number and unit of measurement
☐	☒	A statement on whether measurements were taken from distinct samples or whether the same sample was measured repeatedly
☐	☒	The statistical test(s) used AND whether they are one- or two-sided<br>*Only common tests should be described solely by name; describe more complex techniques in the Methods section.*
☐	☒	A description of all covariates tested
☐	☒	A description of any assumptions or corrections, such as tests of normality and adjustment for multiple comparisons
☐	☒	A full description of the statistical parameters including central tendency (e.g. means) or other basic estimates (e.g. regression coefficient) AND variation (e.g. standard deviation) or associated estimates of uncertainty (e.g. confidence intervals)
☐	☒	For null hypothesis testing, the test statistic (e.g. *F*, *t*, *r*) with confidence intervals, effect sizes, degrees of freedom and *P* value noted<br>*Give P values as exact values whenever suitable.*
☐	☒	For Bayesian analysis, information on the choice of priors and Markov chain Monte Carlo settings
☐	☒	For hierarchical and complex designs, identification of the appropriate level for tests and full reporting of outcomes
☐	☒	Estimates of effect sizes (e.g. Cohen's *d*, Pearson's *r*), indicating how they were calculated

Our web collection on statistics for biologists contains articles on many of the points above.

Software and code

Policy information about availability of computer code

Data collection	We collected 335 Arabidopsis thaliana accessions from the Cape Verde Islands over a series of eight field expeditions between 2012 and 2019, including 189 accessions from 26 stands across four regions in Santo Antão (Cova de Paúl, Lombo de Figueira, Pico da Cruz and Espongeiro), and 146 accessions from 18 stands across three regions in Fogo (Lava, Monte Velha and Inferno). Permits were issued by the Direcção Nacional do Ambiente in Cape Verde (permit no.12/2012, 01/2015, 112/2018).<br>Gridded climate data were downloaded from WorldClim version 2.1 (19 bioclimatic variables), CGAIR-CSI (aridity) and GPS locations of plants were used to extract data in ArcGIS 10.8<br>We sequenced these accessions using Illumina technology.<br>For the experiment under simulated CVI conditions, we used climate data collected in the field sites across years. We collected data on precipitation, soil and air humidity, air temperature, photosynthetically active radiation and light spectrum, using Gemini TinyTag climate data loggers, MultispeQ 2.0 by PhotosynQ, VG-METER-200 by Vegetronix, and Flower Care by Xiaomi.<br>Phenotypic data were manually collected, except for fitness. As a measure of fitness, we counted the total number of seeds produced by taking photographs of them and analyzing those using the Germinator package, implemented in ImageJ v.1.40.
Data analysis	For alignment of short-read data to the reference, SNP identification and calling, we used bcftools v1.9 , bwa v. 0.7.5a, samtools v. 1.9, AdapterRemoval v2.1.2, picard v. 2.21.1, GATK v.4.1.3.0 (identification of SNPs and InDels) and SHORE v0.8 (identification of SNPs)<br>For VCF file format manipulation we used vcftools v. 0.1.14 and bcftools v1.9.<br>To predict effects of polymorphisms we used SnpEff v. 3.0.7.<br>To predict suitable regions for A. thaliana based on Moroccan and Cape Verdean climate, and to produce maps of these, we used MaxEnt 3.4.1 and ESRI ArcGIS<br>To predict fitness effects we used polyDFE v. 2.0.<br>For population structure analysis we used plink v. 1.90b3.45 (file format conversion and NJ tree), Chromopainter v.0.0.4 (estimation of local ancestry across the genome) and R v. 1.2.5033.These sme softwares were used for LD analysis.<br>For demographic modeling we used MSMC v.2 (estimate of population size as a function of time, and of spit times) and dadi v. 2.1 (model-

based demographic inference based on the site frequency spectrum) as well as msprime v.0.4.0 (coalescence simulations) and SLiM v. 3.3.2 (forward simulations) together with custom software in java v. 1.8.
Some of our custom software relied on java v. 1.8 (https://github.com/HancockLab).
For reconstructing histories of FRI and FLC variants we used RELATE v1.1.2 (estimate genealogical trees) and CLUES (estimate selection coefficient).
For quantitative genetic analysis (including GWAS) we used plink v. 1.90b3.45 and GEMMA v. 0.94 (genome-wide association) and R v. 1.2.5033.
All statistical analyses were done using R v.1.2.5033. Specific usage is described in Methods and Supplementary Methods

For manuscripts utilizing custom algorithms or software that are central to the research but not yet described in published literature, software must be made available to editors and reviewers. We strongly encourage code deposition in a community repository (e.g. GitHub). See the Nature Research guidelines for submitting code & software for further information.

Data

Policy information about availability of data

All manuscripts must include a data availability statement. This statement should provide the following information, where applicable:

- Accession codes, unique identifiers, or web links for publicly available datasets
- A list of figures that have associated raw data
- A description of any restrictions on data availability

Data availability statement is included in the mansucript file and reads as follows:
All data generated in this study are included in this article and its Supplementary Materials files. The raw sequencing read data generated in this study have been deposited in the European Nucleotide Archive (ENA) under accession code PRJEB39079 (ERP122550; https://www.ebi.ac.uk/ena/browser/view/PRJEB39079). In addition, previously published sequence data were used from ENA project ID PRJEB24044 (https://www.ebi.ac.uk/ena/browser/view/PRJEB24044) and ENA project ID PRJNA273563 (https://www.ebi.ac.uk/ena/browser/view/PRJNA273563). All sequences were aligned against the Arabidopsis TAIR reference assembly GCA_000001735.1 (ENA; https://www.ebi.ac.uk/ena/browser/view/GCA_000001735.1). The genomic variant calls have been deposited in the European Variation Archive (EVA), under project accession number PRJEB44201 (ERZ1886920; https://www.ebi.ac.uk/ena/data/view/PRJEB44201). Source data are provided with this paper.

Field-specific reporting

Please select the one below that is the best fit for your research. If you are not sure, read the appropriate sections before making your selection.

☒ Life sciences ☐ Behavioural & social sciences ☐ Ecological, evolutionary & environmental sciences

For a reference copy of the document with all sections, see nature.com/documents/nr-reporting-summary-flat.pdf

Life sciences study design

All studies must disclose on these points even when the disclosure is negative.

Sample size	Sample sizes are described in the relevant figure legends. No statistical approach was used to determine sample size in advance of this study, but sizes were based on previously published population genetic and trait-mapping studies. The sample sizes used were adequate to identify significant differences in flowering time and fitness between experimental groups. Samples were selected to maximize variation and thus to achieve a result that represented actual differences between populations.<br>For population genetic clustering analyses, we obtained the maximum possible number of genomes from Morocco and 1001 genomes populations and down-sampled where needed to obtain equal sample numbers among regional groups (for NJ tree and Chromopainter). When computing the joint site frequency spectra for demographic and population genetics analyses, we down-sampled the two CVI populations to 40 samples each and the Moroccan clade to 54. At every site in the genome, accessions were chosen among the ones with non-missing information at the specific site, in order to minimize loss of variants due to missing data.<br>For the phenotyping experiment in the natural populations under CVI conditions, we grew the maximum number of plants possible in our chamber. This included 174 representative accessions from Santo Antão and 129 representative accessions from Fogo, and all sequenced accessions from Morocco (n=64). We also included mutants and controls for specific candidate genes (Col-0, Col-0 FRI-Sf2, and Col-0 FRI-Sf2 flc-3), in four replicates. F2 populations have different sizes (n=488, 636 and 598; as in Methods). For the phenotyping experiment in simulated Moroccan conditions, we used 64 Moroccan accessions, 4 representative accessions from each of the CVI islands, all in 4 replicates. For the RNA expression experiment, three replicates for each genotype were used. For the complementation test experiment, we phenotyped 4 replicates of each parental line (Col-0 FRI-Sf2 flc-3, F3-2, F9-2, F13-8, F10-1-3) and 8 of each F1.
Data exclusions	No data were excluded from the initial set of 335 accessions, nor from the phenotypic experiments.
Replication	For population genetic clustering analyses (NJ tree, Chromopainter), we repeatedly down-sample each genetic cluster to a uniform sample size in order to determine repeatability within the data set.<br>For demographic inference with MSMC v.2, we used a total of 63 combinations of eight accessions from each island. For the split to Morocco, we used 357 combinations (separately for the High Atlas, South and North Middle Atlas Moroccan populations). For splits within Santo Antão, we used 12 combinations. We used different combinations of sets of samples in order to determine repeatability within the data set.<br>For demographic inference with dadi, we identified the model with the best support using the Akaike information criterion (AIC), and for each resulting best model, we calculated confidence intervals for parameters using 100,000 bootstrapped data sets and the Godambe Information Matrix.<br>For the dNeu/dSel and DFE analyses, we bootstrapped the data 500 times for each population, in order to quantify the spread around the point estimates for these statistics.<br>In the phenotyping experiment under simulated CVI conditions, accessions from Cape Verde natural populations were grown in four replicates

	and accessions from Morocco two replicates. For mutants, controls and other experiments, we grew all lines in four replicates, except in specific cases explained in Methods and Supplementary Methods.
Randomization	In all experiments plants were organized in randomized block designs.
Blinding	The experiment was not conducted blindly. However, genotypes were randomized and fitness data were collected by assistants who did not have knowledge of the specific hypotheses being tested. It was not possible to blind groups for data analysis, but blinding in this context was not relevant because we used standard analysis pipelines with predetermined hypotheses.

Reporting for specific materials, systems and methods

We require information from authors about some types of materials, experimental systems and methods used in many studies. Here, indicate whether each material, system or method listed is relevant to your study. If you are not sure if a list item applies to your research, read the appropriate section before selecting a response.

Materials & experimental systems

n/a	Involved in the study
☒	☐ Antibodies
☒	☐ Eukaryotic cell lines
☒	☐ Palaeontology and archaeology
☒	☐ Animals and other organisms
☒	☐ Human research participants
☒	☐ Clinical data
☒	☐ Dual use research of concern

Methods

n/a	Involved in the study
☒	☐ ChIP-seq
☒	☐ Flow cytometry
☒	☐ MRI-based neuroimaging
